# The cytoprotective protein clusterin is overexpressed in hypergastrinemic rodent models of oxyntic preneoplasia and promotes gastric cancer cell survival

**DOI:** 10.1371/journal.pone.0184514

**Published:** 2017-09-13

**Authors:** Pål Vange, Torunn Bruland, Berit Doseth, Reidar Fossmark, Mirta M. L. Sousa, Vidar Beisvag, Øystein Sørdal, Gunnar Qvigstad, Helge L. Waldum, Arne K. Sandvik, Ingunn Bakke

**Affiliations:** 1 Department of Clinical and Molecular Medicine, NTNU, Norwegian University of Science and Technology, Trondheim, Norway; 2 Clinic of Medicine, St. Olav’s University Hospital, Trondheim, Norway; 3 Department of Gastroenterology and Hepatology, St. Olav’s University Hospital, Trondheim, Norway; 4 Centre of Molecular Inflammation Research (CEMIR), NTNU, Trondheim, Norway; Istituto di Ricovero e Cura a Carattere Scientifico Centro di Riferimento Oncologico della Basilicata, ITALY

## Abstract

The cytoprotective protein clusterin is often dysregulated during tumorigenesis, and in the stomach, upregulation of clusterin marks emergence of the oxyntic atrophy (loss of acid-producing parietal cells)-associated spasmolytic polypeptide-expressing metaplasia (SPEM). The hormone gastrin is important for normal function and maturation of the gastric oxyntic mucosa and hypergastrinemia might be involved in gastric carcinogenesis. Gastrin induces expression of clusterin in adenocarcinoma cells. In the present study, we examined the expression patterns and gastrin-mediated regulation of clusterin in gastric tissue from: humans; rats treated with proton pump (H+/K+-ATPase) inhibitors and/or a gastrin receptor (CCK2R) antagonist; H+/K+-ATPase β-subunit knockout (H/K-β KO) mice; and Mongolian gerbils infected with *Helicobacter pylori* and given a CCK2R antagonist. Biological function of secretory clusterin was studied in human gastric cancer cells. Clusterin was highly expressed in neuroendocrine cells in normal oxyntic mucosa of humans and rodents. In response to hypergastrinemia, expression of clusterin increased significantly and its localization shifted to basal groups of proliferative cells in the mucous neck cell-chief cell lineage in all animal models. That shift was partially inhibited by antagonizing the CCK2R in rats and gerbils. The oxyntic mucosa of H/K-β KO mice contained areas with clusterin-positive mucous cells resembling SPEM. In gastric adenocarcinomas, clusterin mRNA expression was higher in diffuse tumors containing signet ring cells compared with diffuse tumors without signet ring cells, and clusterin seemed to be secreted by tumor cells. In gastric cancer cell lines, gastrin increased secretion of clusterin, and both gastrin and secretory clusterin promoted survival after starvation- and chemotherapy-induced stress. Overall, our results indicate that clusterin is overexpressed in hypergastrinemic rodent models of oxyntic preneoplasia and stimulates gastric cancer cell survival.

## Introduction

In the gastric oxyntic mucosa, glands are divided into different zones containing characteristic cell lineages that normally differentiate from immature progenitor cells in isthmus [1–3]. During carcinogenesis, the typical differentiation pattern is disrupted and the mucosa undergoes step-wise transformation, which for the intestinal type gastric adenocarcinoma is thought to progress through oxyntic atrophic (loss of acid-secreting parietal cells) gastritis, intestinal metaplasia and dysplasia before emergence of cancer [4, 5]. In addition, spasmolytic polypeptide-expressing metaplasia (SPEM), which possibly evolves by transdifferentiation of mature chief cells, may develop prior to intestinal metaplasia and play a central role in the early phases of the cascade [6–8].

Gastrin is a key secretagogue for gastric acid, and regulates cell proliferation, apoptosis and migration, making it essential for normal growth and maturation of the oxyntic mucosa [9–11]. Hypergastrinemia might promote gastric carcinogenesis, particularly when combined with oxyntic atrophy and chronic inflammation due to *Helicobacter*-infection [11, 12]. The pro-survival cytoprotective protein clusterin (CLU) is gastrin-responsive in rat pancreatic adenocarcinoma cells, and is involved in the anti-apoptotic effect of gastrin [13]. In oxyntic mucosa of rats, proton pump (H+/K+-ATPase) inhibitor (PPI)-induced hypergastrinemia led to increased CLU expression and a prominent shift in the CLU expression pattern [13].

CLU is nearly ubiquitously expressed in different tissues and associated with regulation of cell survival, migration/invasion, differentiation, cellular stress responses, and resistance to cancer therapy [14, 15]. The secretory CLU (sCLU) isoform is characterized as a stress-responsive extracellular chaperone with functional similarities to small heat shock proteins [14, 16]. Dysregulation of CLU is found during malignant progression in several tissues and might follow a dysplasia-dependent U-shaped curve [14, 17]: in normal tissues, CLU can inhibit tumorigenesis by sensing and counteracting cellular stress [18], while dysplastic cells can temporarily downregulate CLU expression to allow malignant transformation [14]. Then, in established malignancies, upregulation of CLU can promote anti-apoptotic signaling, resistance to chemotherapy, and increased metastatic spread [14, 16, 18].

CLU has also been linked to gastric cancer in some studies [17, 19–24]. Aberrant expression in cell cultures, rodent models, and human specimens containing SPEM, suggests that CLU is a marker of SPEM [17, 23, 25]. Studies show conflicting results regarding the value of CLU in blood as a biomarker of gastric cancer [20–22, 24]. Nevertheless, overexpression of CLU in gastric cancer biopsies is reported to correlate with lymph node metastasis, tumor invasion, and high tumor stage [17, 19, 21].

Still, expression and function of CLU in normal gastric physiology, hypergastrinemic conditions and during gastric carcinogenesis have not been widely examined. In the present study, we characterize the expression and regulation of CLU in gastric oxyntic mucosa of hypergastrinemic rodent models and in humans, and elucidate the function of sCLU in human gastric cancer cells during stress.

## Materials and methods

### Animal studies

Animal studies were approved by the Norwegian Animal Research Authority (Approval no. 5014) and performed according to international guidelines. All animals were housed in plastic cages under standard conditions of 20°C, 40–45% relative humidity, 12-hours light-dark cycle, and free access to commercial chow and water. The PPI pantoprazole (400 μmol/kg animal weight) (Nycomed, Konstanz, Germany) was administered daily by gavage to female Sprague-Dawley rats (150–200 g) (Taconic M&B, Skensved, Denmark) for 3 weeks. The gastrin receptor/cholecystokinin receptor 2 (CCK2R) antagonist netazepide (YF476) (500 μmol/kg animal weight) (kindly provided by Dr. M. Boyce, Hammersmith Medicines Research, London, UK) was given as a single dose treatment, and was injected subcutaneously at day 1. Groups of rats received combined treatment with pantoprazole and netazepide (n = 8), pantoprazole (n = 6) or netazepide (n = 8) alone, or only vehicle as control (n = 9).

Formalin-fixed paraffin-embedded (FFPE) biopsies from oxyntic mucosa of male and female H+/K+-ATPase (proton pump) β-subunit knockout (H/K-β KO) mice aged 3, 6, 8 and 14 months and BALB/c wild-type mice aged 8 months as controls (n = 4 per age group), were as described [26]. FFPE biopsies from oxyntic mucosa of male Mongolian gerbils were from three groups, as previously described [27]: infected with *Helicobacter pylori* (n = 7) for 18 months; infected with *H*. *pylori* and treated with netazepide for 18 months (n = 7); and uninfected control animals aged 12 months (n = 5).

### Human tissues

FFPE biopsies of human gastric mucosa were from specimens gathered immediately after gastrectomy from 59 patients (35 male/24 female, mean age 66.5 years (range 45–98)) at St. Olav’s University Hospital, Trondheim, Norway. Adjacent non-tumor tissue was collected from 21 patients (18 male/3 female, mean age 65.9 years (range 49–86)). A pathologist diagnosed all patients histologically as primary gastric adenocarcinoma of TNM stage 0/IA (n = 1), IA/IB (n = 7), II/IIIA/IIIB (n = 38), IV (n = 11), and unknown (n = 2). Of these, 30 were of the Laurén intestinal type localized in antrum (n = 12), corpus (n = 8) or cardia (n = 10), 19 were of the diffuse type localized in antrum (n = 6), corpus (n = 3) or cardia (n = 10), and 10 were of the diffuse type containing signet ring cells (SRCs) localized in antrum (n = 7), corpus (n = 2) or cardia (n = 1). In addition, 16 matched normal mucosa specimens (13 male /3 female, mean age 73.0 years (range 52–82)) from patients with no signs of gastric neoplasm were collected. Collection and use of patient material were after written informed consent and approval by the Regional Committee for Medical and Health Research Ethics of Central Norway (Approval no. 018–02.).

### Gene expression analysis of clusterin in human gastric adenocarcinomas

The RNA isolation and microarray analysis of the expression profile of *CLU* mRNA followed standard protocols, analyzing 300 ng total RNA per sample with the HumanHT-12 Expression BeadChips (Illumina, San Diego, CA) (ArrayExpress E-MTAB-1338). Analyses of *CLU* mRNA expression in human gastric adenocarcinomas were done using our in-house dataset and the Oncomine database (www.oncomine.org), as previously described [3].

### Human gastric cancer cell lines

The following human gastric cancer cell lines were used: AGS wild-type (AGSwt) (American Type Culture Collection (ATCC) Rockville, MD) (negative control for gastrin-induced changes), AGS stably transfected with CCK2R (AGS-GR) (provided by Prof. Andrea Varro, University of Liverpool, Liverpool, UK), MKN-45 (gift from Queens Medical Centre, University Hospital, Nottingham, UK) and KATO-III (ATCC). AGSwt and AGS-GR were grown in HAM’S F12 (GIBCO, Invitrogen, Carlsbad, CA) with 10% fetal calf serum (FCS), 10 U/ml penicillin-streptomycin, and 2 μg/ml puromycin (Sigma-Aldrich, St. Louis, MO). KATO III was grown in RPMI (GIBCO, Invitrogen) with 20% FCS, 10 U/ml penicillin-streptomycin, 1 μg/ml fungizone (GIBCO, Invitrogen) and 0.1 mg/ml L-Glutamine added. MKN45 was grown in DMEM (GIBCO, Invitrogen) with 4.5 g/l glucose, 10% FCS, 1 mM sodium pyruvate, 0.1 mg/ml L-glutamine, 10 U/ml penicillin-streptomycin, and 1 μg/ml fungizone (GIBCO, Invitrogen). All cell lines were kept in 75 cm^2^ flasks incubated at 37°C, 5% CO2 and 95% air.

### Immunohistochemistry

The primary antibodies used are listed in Table 1. For immunohistochemistry (IHC), standard pretreatments of FFPE sections (4 μm), including deparaffinization, rehydration, and antigen retrieval with boiling in citrate buffer (pH 6.0) using a commercial microwave, were followed by incubation with the primary antibodies for 2 hours at room temperature (RT) or 4°C overnight. Single immunoreactions were visualized using the rabbit/mouse EnVision-horseradish peroxidase (HRP)/DAB+ kit (#K4003 and #K5007, Dako, Glostrup, Denmark) or Vectastain Elite ABC HRP Kit (Elite PK-6100 Standard, Vector Laboratories Inc., Burlingame, CA) and counterstained with hematoxylin. For immunofluorescence staining, primary antibodies were incubated simultaneously and visualized using secondary antibodies conjugated to Alexa Fluor 488, 555 or 647 (Invitrogen) 1:200 or 1:400 dilution, or lectin *Griffonia simplicifolia* (GSII) (Life Technologies, Grand Island, NY) 1:400 dilution, and counterstained with DAPI. The primary antibodies rabbit anti-CLU (H-330, sc-8354, Santa Cruz Biotechnology Inc., Dallas, TX) and rabbit anti-pepsinogen 5 (PGA5) (17330-1-AP, Proteintech Europe, Manchester, United Kingdom) were detected with tyramide signal amplification^™^ PLUS Fluorescence Kit (NEL760001KT, Perkin Elmer, Waltham, MA) according to manufacturer’s instructions. In addition to omitting the primary antibody, non-immunized rabbit, mouse or goat IgG at similar protein concentration as the primary antibody, were used as negative controls.

**Table 1:** Primary antibodies used in immunofluorescence staining and immunohistochemistry.

Name	Host, clonality	Clone or catalog no; Source	Dilution		Incubation	Lineage marker
			IF 1:	IHC 1:		
Clusterin	Rabbit, P	H-330, sc-8354; Santa Cruz Biotechnology Inc., Dallas, TX	150	150	2 h RT / 4°C on	
Clusterin-α	Goat, P	C-18, sc-6419; Santa Cruz Biotechnology Inc.	500	1000	2 h RT / 4°C on	
Clusterin-α	Mouse, M	B-5, sc-5289; Santa Cruz Biotechnology Inc.	500		2 h RT / 4°C on	
H+/K+ ATPase β	Mouse, M	2G11, MA3-923; Affinity Bioreagent Inc, Golden, CO	1500		2 h RT / 4°C on	Parietal cells
Mist 1/BHLHA15	Mouse, M	6E8, sc-80984; Santa Cruz Biotechnology Inc.	200		4°C on	Chief cells
Pepsinogen 5, group I (pepsinogen A)	Rabbit, P	17330-1-AP; Proteintech Europe, Manchester, UK	50		4°C on	Chief cells
Pepsinogen II	Sheep, P	Ab9013; Abcam, Cambridge, UK	300		2 h RT / 4°C on	Chief cells
Lectin GS-II, A488 conj.	N/A	L21415; Molecular probes, Grand Island, NY	200		2 h RT	Neck cells, SPEM
Trefoil factor 2	Rabbit, P	13681-1-AP; Proteintech Europe	300	900	2 h RT / 4°C on	Neck cells, SPEM
Chromogranin A	Rabbit, P	SP-1, 20086; Immunostar, Hudson, WI	1500		2 h RT	Neuroendocrine
Human chromogranin A	Mouse, M	M0869, clone DAK-A3; Dako, Glostrup, Denmark	1000		4°C on	Neuroendocrine
Histidine decarboxylase	Rabbit, P	B 260–1; Eurodiagnostica, Malmö, Sweden	1000	3000	2 h RT / 4°C on	ECL cells
Vesicular monoamine transporter 2	Rabbit, P	AB1767; Chemicon, Temecula, CA	500		2 h RT / 4°C on	ECL cells
Ghrelin	Rabbit, P	H-031-31; Phoenix Pharmaceuticals Inc., Burlingame, CA	7000		2 h RT	A-like cells
Proliferating cell nuclear antigen	Mouse, M	M0879, clone PC10; Dako	200		4°C on	Proliferation
Ki67	Rabbit, P	Ab15580; Abcam	300		4°C on	Proliferation
Mucin 2	Rabbit, P	Ab76774; Abcam		200	2 h RT	Goblet cells, IM

Abbreviations: P = polyclonal, M = monoclonal, IF = immunofluorescence staining, IHC = immunohistochemistry, h = hours, RT = room temperature, on = overnight, SPEM = spasmolytic polypeptide-expressing metaplasia, IM = intestinal metaplasia

### *In situ* hybridization

*In situ* hybridization (ISH) was performed with the RNAscope 2.0 HD Reagent Kit (Brown) for FFPE tissue (310035, Advanced Cell Diagnostics (ACD) Inc., Hayward, CA) according to manufacturer’s instructions. After pretreatment, sections were incubated with custom transcript- and species-specific probes against clusterin mRNA for 2 hours in a humid chamber at 40°C, followed by a series of amplifications and visualization with HRP and DAB. Sections were counterstained with hematoxylin. Target-specific probes against bacterial RNA (DapB) and ubiquitin C were used as negative and positive controls, respectively.

### Periodic acid Schiff and Alcian blue staining

Tissue sections of FFPE biopsies from control and H/K-β KO mice were incubated in Alcian blue solution (B8438, Sigma-Aldrich) for 5 minutes, periodic acid solution for 10 minutes, Schiff’s reagent (101646, Merck Millipore, Darmstadt, Germany) for 15 minutes and then hematoxylin for 3 minutes.

### Assay of gastrin and CLU concentrations in rat plasma

Gastrin concentrations were measured using radioimmunoassay as previously described [28] and CLU protein in plasma was quantified using Rat CLU ELISA kit (BioVendor Laboratorni Medicina AS, Modrice, Czech Republic) according to instructions.

### Sample preparation for targeted mass spectrometry analysis

Proteins were extracted for targeted mass spectrometry from rat oxyntic mucosa lysates and protein concentrations measured, as previously described [29]. 50 μg protein of each lysate were incubated in 5 mM tris (2-carboxyethyl) phosphine (TCEP) for 30 min at RT followed by alkylation with iodoacetamide (1 μmol/mg protein) for 30 min in the dark. Proteins were precipitated using a methanol-chloroform method as described [30] and submitted to another round of protein reduction and alkylation by resuspension in 50 μl 50 mM NH_4_HCO_3_, 5 mM TCEP, incubation for 30 min and subsequent incubation with 1 μmol/mg protein of iodoacetamide for 30 min in the dark. Trypsin (Thermo Scientific, Waltham, MA) was added at 1:50 ratio (w/w, enzyme:protein) prior to overnight digestion at 37 ^◦^C in a shaker. Subsequently, formic acid was added to all samples (final concentration 0.1%) followed by centrifugation for 10 minutes at max. speed (16 000 g) for removal of insoluble particles prior to mass spectrometry analysis.

### Targeted mass spectrometry

All parallel reaction monitoring (PRM)-based targeted mass spectrometry methods were designed, analyzed, and processed using Skyline software version 3.6.0.10162 [31]. *In silico* selection of proteotypic peptides was performed via Skyline using the *Rattus norvegicus* reference proteome available at www.uniprot.org to exclude non-unique peptides. Synthetic light peptides (Thermo Scientific) were used as standards for targeted mass spectrometry analysis. Peptide standards were first analyzed on a Thermo Scientific Q Exactive HF mass spectrometer coupled to an Ultimate 3000 RSLC system (Thermo Scientific, Sunnyvale, California, USA) in PRM mode. Precursor ions of higher intensity (charge state 2+ or 3+) were selected for further analysis. Information on retention time and fragmentation pattern of the standard peptides was used for identification and to build a scheduled method with a retention time window of 5 min. The method was then employed for detection and quantification of corresponding peptides in rat samples. The same instrument parameters described below for the analysis of rat samples were adopted for the establishment of the PRM method with standard peptides.

Peptides (2 μg) were separated during a biphasic ACN gradient from two nanoflow UPLC pumps (flow rate of 200 nL/min) on a Acclaim PepMap100 C18 column (75 μm i.d. × 2 cm nanoviper, 3 μm particle size, 100 Å pore size) (Thermo Scientific) and further separated on a PepMap RSLC C18 analytical column (50cm x 75 μm i.d. EASY-spray column, packed with 2μm C18 beads) (Thermo Scientific). Solvent A and B were 0.1% TFA (vol/vol) in water and 100% ACN respectively. The gradient composition was 5%B for 5 min followed by 5–8%B over 0.5 min, 8–24%B for the next 109.5 min, 24–35%B over 25 min, and 35–90%B over 15 min. Elution of very hydrophobic peptides and conditioning of the column were performed during 15 minutes isocratic elution with 90%B and 20 min isocratic conditioning with 5%B.

The peptides eluting LC-column were ionized in the electrospray and analyzed by the Q-Exactive HF in Parallel Reaction Monitoring (PRM) mode. The spray and ion-source parameters were as follows. Ion spray voltage = 1800V, no sheath and auxiliary gas flow, and capillary temperature = 250°C. Instrument control was through Q Exactive HF Tune 2.4 and Xcalibur 3.0 MS spectra were acquired in the scan range 375–1500 m/z with resolution R = 15,000 at m/z 200, automatic gain control (AGC) target of 3e6 in and maximum injection time (IT) of 15ms. Target MS2 spectra (Top15) were acquired with a resolution R = 15,000, AGC target of 1e5, IT of 100 ms and normalized collision energy of 28%. The isolation window was set to 1.6 m/z for the selection of the precursor. Lock-mass internal calibration was used.

Quantification of peptides detected in rat samples was achieved by summing the integrated peak areas of the most intense fragments. Peptide areas for multiple peptides of the same protein were summed to assign relative abundance to that protein. A minimum of two peptides per protein was used for quantification. Endogenous β-actin levels were used for data normalization.

### Expression and secretion of CLU in human gastric cancer cells

AGS-GR cells were treated with gastrin (Gastrin 17) (G9020, Sigma-Aldrich) 5 and 10 nM or cisplatin (479306, Sigma-Aldrich) 10 or 20 μM for 24 or 48 hours, and conditioned medium and whole cell lysates were harvested.

For western blot, proteins were extracted from cell lines as previously described [29]. Whole cell extracts (60 μg protein/well) were separated on NuPAGE 4–12% Bis-Tris gels (Invitrogen) and electroblotted onto Immobilon PVDF membranes (Millipore, Billerica, MA). The membranes were blocked with 5% BSA in PBS-Tween, and incubated overnight at 4°C with mouse anti-CLU (B-5, sc-5289, Santa Cruz Biotechnology Inc.) 1:800 and rabbit anti-β-tubulin (ab6046, Abcam, Cambridge, United Kingdom) 1:5000, diluted in 1% BSA in PBS-Tween. After washing, the membranes were further incubated for 1 hour at RT with HRP-conjugated rabbit anti-mouse IgG (P0260, Dako) 1:5000 or HRP-conjugated swine anti-rabbit IgG (PO399, Dako) 1:5000. Binding of antibodies was developed using SuperSignal West Femto Maximum Substrate and visualized on LI-COR’s Odyssey-Mode imaging system. Prior to western blot analysis of secreted protein, conditioned cell culture media was concentrated from 500 μL to 70 μL using Amicon^®^ ultra-0.5 centrifugal filter devices (10,000 NMWL) according to the manufacturer’s recommendations.

For immunocytochemistry, serum-starved (7 hours) AGS-GR cells were treated with gastrin (G9020, Sigma-Aldrich) 10 nM for 24 or 48 hours, before they were washed with cold PBS and fixed 20 minutes at RT using freshly made 3.7% paraformaldehyde + 4% sucrose in PBS. Following permeabilization and blocking of unspecific binding, cells were incubated with rabbit anti-CLU (H-330, sc-8354, Santa Cruz Biotechnology Inc.) 1:50 dilution at 4°C overnight, followed by re-blocking. Then incubation with secondary antibody goat anti-rabbit A488 (A11008, Invitrogen) diluted 1:400 was done for 60 minutes at RT. Lastly, nucleic DNA and actin was sequentially stained with DAPI (D3571, Invitrogen) (0.1 μg/ml in PBS) and then Rhodamine Phalloidin (R415, Invitrogen) diluted 1:100 both for 5 minutes at RT, and replaced with PBS and kept in the dark at 4°C. Non-immunized rabbit IgG or omitting the primary antibody, were used as negative controls.

### Migration assay

xCelligence migration assay (Roche, Basel, Switzerland) was performed according to the manufacturer’s instructions with some minor modifications. AGS-GR and AGSwt cells were split 1:2 the day before the experiment and seeded out at 5 × 10^4^ cells per well in a 16-well CIM-plate (Roche). Cells were treated with serum-free media or gastrin (G9020, Sigma-Aldrich) 0.1 to 10 nM. Gastrin-induced migration was confirmed using a scratch-assay [32]. AGSwt cells were used as negative control. Goat anti-CLU (C-18, sc-6419, Santa Cruz Biotechnology Inc.) 8 μg/ml (previously used to neutralize sCLU [13, 33]), non-immunized goat polyclonal IgG 8 μg/ml or recombinant sCLU (Clusterin Human HEK293, RD172034100, BioVendor, Brno, Czech Republic) 50 nM or 200 nM were added. Analysis of migration was performed after 18 hours.

### Survival assay

Caspase Glo^®^ 3/7 Assay (Promega Corporation, Fitchburg, WI) was performed according to manufacturer’s instructions. Apoptosis was induced by 48 hours of serum-starvation. AGS-GR cells were treated with gastrin (G9020, Sigma-Aldrich) 5 or 10 nM and combinations of goat anti-CLU (C-18, sc-6419, Santa Cruz Biotechnology Inc.) 8 μg/ml, non-immunized goat polyclonal IgG 8 μg/ml, recombinant sCLU (Clusterin Human HEK293, RD172034100, BioVendor) 200 nM and cisplatin (479306, Sigma-Aldrich) 10 μM.

TiterTACS In Situ Detection Kit—Colorimetric (4822-96-K, R&D Systems, Minneapolis, MN) allows terminal deoxynucleotidyl transferase dUTP nick end labeling (TUNEL) staining of cells in a 96-well format, and was performed according to manufacturer’s instructions. Apoptosis was induced by 72 hours of serum-starvation. AGS-GR cells were treated with gastrin (G9020, Sigma-Aldrich) 10 nM and/or recombinant sCLU (Clusterin Human HEK293, RD172034100, BioVendor) 200 nM.

### Morphometrics, statistics and imaging

Enterochromaffin-like (ECL) cell hyperplasia was defined as presence of histidine decarboxylase (HDC)-positive cells in linear or micronodular patterns [34]. The total number of CLU-positive cells and the number of CLU-positive cells also expressing PGA5, vesicular monoamine transporter 2 (VMAT2) or proliferating cell nuclear antigen (PCNA) was counted in >40 glands per rat (n = 4–8 per group). The number of CLU-positive cells also expressing Ki67 or VMAT2 was counted in similar basal mucosal areas (0.44 mm^2^) in control and H/K-β KO mice of all ages (n = 4 per group) (Ki67) and all Mongolian gerbils (n = 4–7 per group) (Ki67 and VMAT2). All dual CLU- and chromogranin A (CgA)-positive cells were counted in pinch biopsies (n = 9) of normal human gastric mucosa. To calculate relative migration and apoptosis ratios, results from xCelligence and Caspase assays were normalized to median of the untreated control (or non-immunized goat IgG) in each individual experiment. Statistically significant differences (*p* value<0.05) were analyzed using analysis of variation (ANOVA) with Bonferroni’s or Tukey’s multiple comparison test or Student’s t-test using Prism 7 (GraphPad Software, San Diego, CA) and Microsoft Excel 2013 (Redmond, WA). Chromogenic images were captured using Nikon E400 microscope, DS-Fil U2 camera and NIS-Elements BR imaging software (Nikon Co., Tokyo, Japan). Immunofluorescent images of tissue were captured using Olympus IX71 inverted microscope, digital monochrome XM10 camera and P^cell software (Olympus Co. Tokyo, Japan) and further processing using ImageJ (Wayne Rasband, National Institutes of Health, USA), and of cells with Leica SP8 inverted microscope (LeicaMicrosystems, Mannheim, Germany) equipped with an HC PL APO 63x/1.20 W and further processing using Fiji [35].

## Results

### Neuroendocrine cells in normal oxyntic mucosa express clusterin

**Fig 1:**
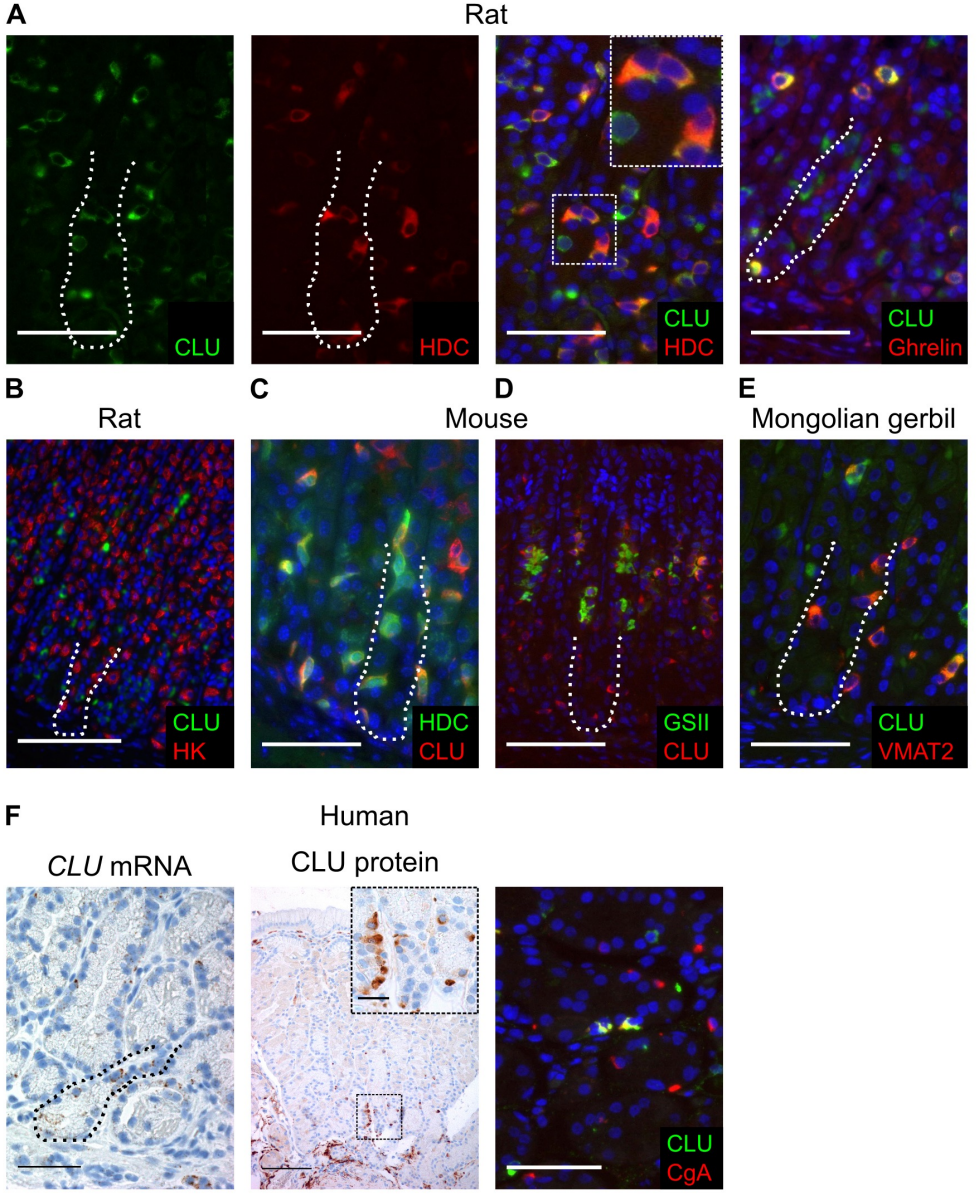
Clusterin expression in neuroendocrine cells. (A) Double immunofluorescence staining of oxyntic mucosa from control rats showing CLU (green) expression in ECL cells (HDC-positive (red)) and A-like cells (ghrelin-positive (red)). A split image of the green (CLU) and red (HDC) fluorescences separately, and a higher magnification of a few dual positive cells (inset), are shown to clearly illustrate the co-expression of CLU in neuroendocrine cells, representative for all species. (B) Double immunofluorescence staining of oxyntic mucosa from control rats showing no CLU (green) expression in parietal cells (H+/K+-ATPase β (HK)-positive (red)). (C) Double immunofluorescence staining of oxyntic mucosa from wild-type control mice showing CLU (red) expression in ECL cells (HDC-positive (green)). (D) Double immunofluorescence staining of oxyntic mucosa from wild-type control mice showing CLU (red) expression in mucous neck cells (GSII-positive (green)). (E) Double immunofluorescence staining of oxyntic mucosa from control Mongolian gerbils showing CLU (green) expression in ECL cells (VMAT2-positive (red)). (F) ISH and IHC of human oxyntic mucosa showing clusterin (brown and green) expression in scattered single cells partially overlapping with the neuroendocrine marker CgA (red). Inset shows high power view of single CLU-positive cells. Nuclei were counterstained with hematoxylin (blue) or DAPI (blue). The basal zone (~100 μm from the gland bottom) is highlighted with a dotted line. Scale bars = (B, D, F middle column) 100 μm; (A, C, E, F left and right column) 50 μm; (F inset) 20 μm.

We have previously indicated using serial section staining that neuroendocrine cells (presumably ECL cells) in normal rat oxyntic mucosa and human carcinoids express CLU [13]. Here, we use double immunofluorescence staining, with antibodies against CLU and known markers for different neuroendocrine cell types, to confirm and further elucidate CLU expression in neuroendocrine cells in normal oxyntic mucosa from three different rodent models and humans (Fig 1). In rat oxyntic mucosa, the single cells expressing high levels of CLU were ECL cells (HDC-positive) and A-like cells (ghrelin-positive) (Fig 1A). In oxyntic mucosa of wild-type mice, CLU expression pattern was different from other rodents, with CLU expressed in ECL cells (HDC-positive) (Fig 1C) as well as in mucous neck cells (GSII-positive) (Fig 1D), as previously reported [17]. Also, in oxyntic mucosa of normal Mongolian gerbils, CLU was expressed mainly in ECL cells (VMAT2-positive) (Fig 1E). In human oxyntic mucosa, the CLU mRNA and protein expression patterns were similar to rodents, with highly CLU-positive single cells partly co-expressing the neuroendocrine cell marker CgA (26.5%), and other gland cells showing more diffuse expression (Fig 1F). There was no CLU expression in parietal cells in any of the examined species (Fig 1B and S1A Fig). Taken together, these results show that, in normal oxyntic mucosa in different species, neuroendocrine cells, particularly ECL cells, express high levels of CLU; in addition, there is less prominent expression of CLU in cells of the mucous neck cell-chief cell lineage.

### Gastrin/CCK2 receptor signaling contributes to the regulation of clusterin expression in oxyntic mucosa of rats

**Fig 2:**
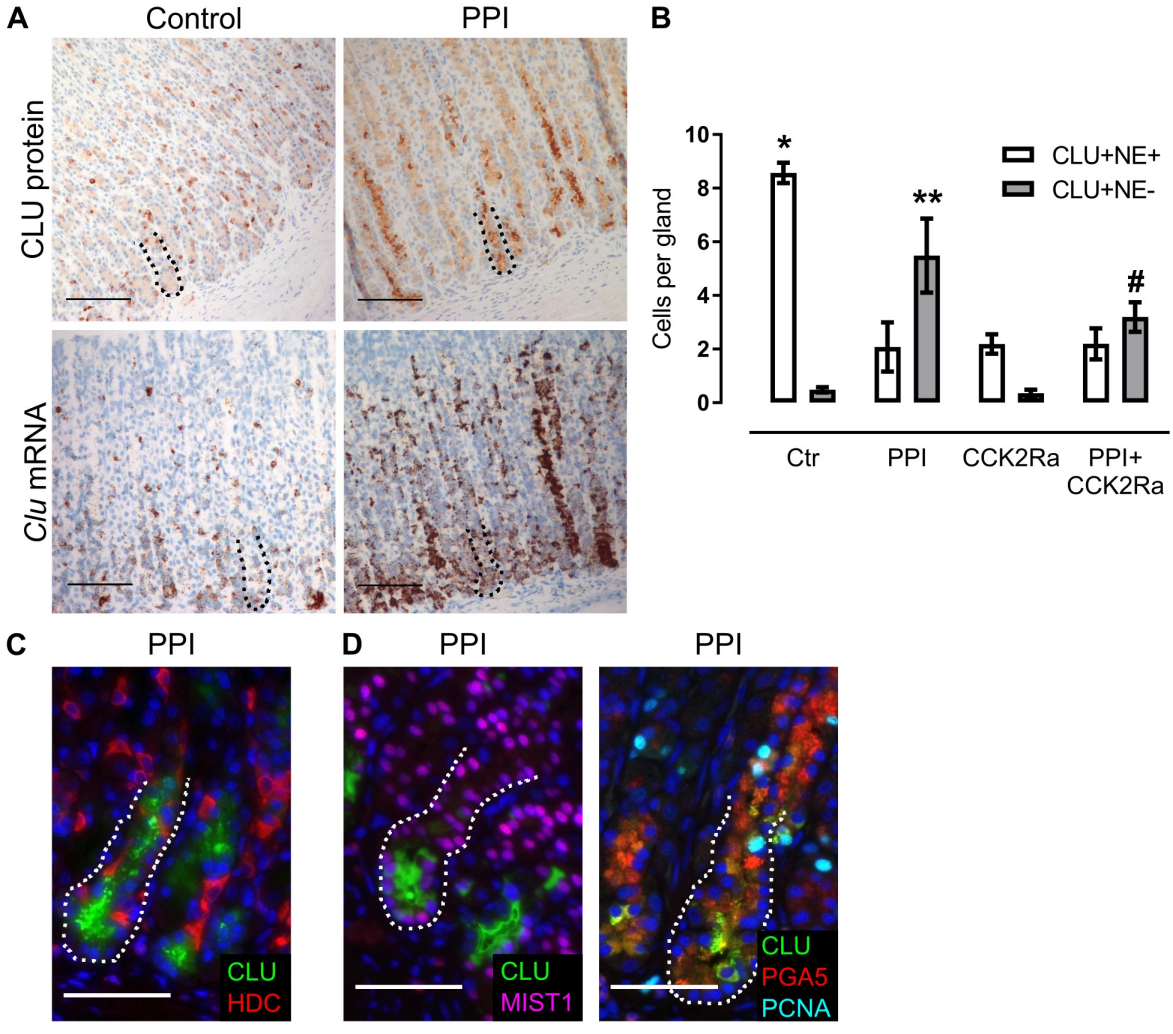
Clusterin expression in oxyntic mucosa increases and localization shifts from neuroendocrine cells to chief cells after sustained hypoacidity and hypergastrinemia. (A) IHC and ISH showing similar expression patterns for clusterin (brown) protein and mRNA, examined in control (n = 8) and hypergastrinemic PPI-rats (n = 6). (B) Number of CLU-positive/VMAT2(neuroendocrine (NE))-positive cells and CLU-positive/VMAT2(NE)-negative cells per oxyntic gland (n = 4 rats per group). (C) Double immunofluorescence staining of oxyntic mucosa from hypergastrinemic PPI-rats showing scarce CLU (green) expression in ECL cells (VMAT2-positive (red)). (D) Double immunofluorescence staining of oxyntic mucosa from hypergastrinemic PPI-rats showing CLU (green) expression in groups of chief cells (MIST1-positive (purple)) and triple immunofluorescence staining showing CLU (green) expression in proliferating (PCNA-positive (light blue)) chief cells (PGA5 (red)). Ctr = control; PPI = PPI-induced hypergastrinemia; CCK2Ra = CCK2R antagonist; PPI+CCK2Ra = PPI-induced hypergastrinemia+CCK2R antagonist. Data presented as means ± SEM. *, ** and # = ANOVA with Bonferroni-adjusted *p* value < 0.05. (*comparison of CLU+/NE+ cells per gland in control vs other groups individually; **comparison of CLU+/NE- cells per gland in PPI vs control or CCK2Ra; #comparison of CLU+/NE- cells per gland in PPI+CCK2Ra vs CCK2Ra.) Nuclei were counterstained with hematoxylin (blue) or DAPI (blue). The basal zone (~100 μm from the gland bottom) is highlighted with a dotted line. Scale bars = (A) 100 μm; (C, D) 50 μm.

**Fig 3:**
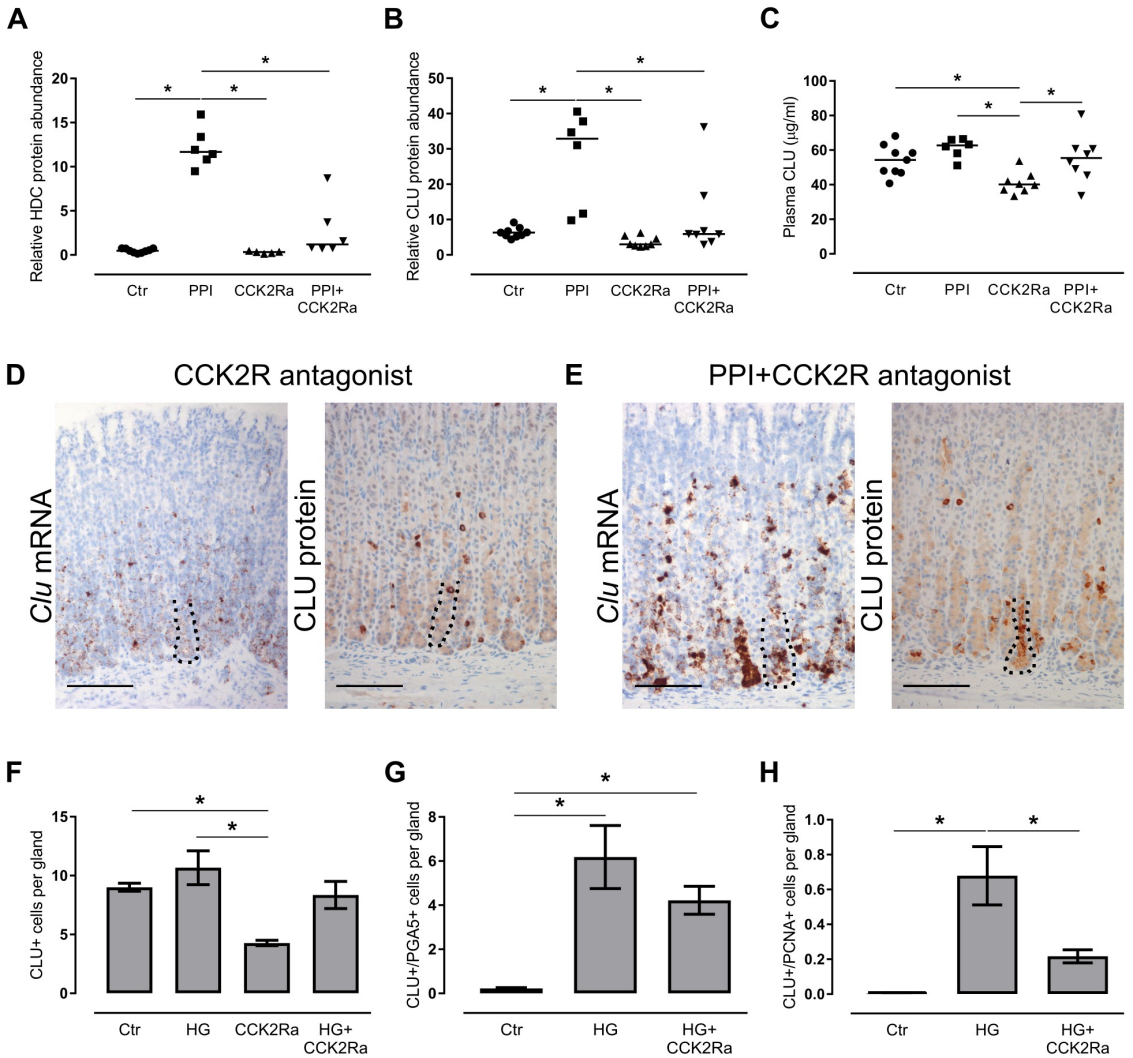
Clusterin expression is regulated by CCK2R signaling and suppression of gastric acid secretion. (A, B, C) HDC protein level (A) and CLU protein level (B) in whole mucosa lysates by targeted mass spectrometry and plasma (C) by ELISA from control (Ctr) (n = 9), PPI-induced hypergastrinemic (PPI) (n = 6), CCK2R-antagonized (CCK2Ra) (n = 5–8) and CCK2R-antagonized PPI-induced hypergastrinemic (PPI+CCK2Ra) (n = 6–8) rats. Each dot represents an individual animal and the black lines mark median. (D) ISH (n = 4) and IHC (n = 8) of oxyntic mucosa from CCK2R-antagonized rats showing expression of clusterin (brown). (E) ISH (n = 5) and IHC (n = 8) of oxyntic mucosa from CCK2R-antagonized hypergastrinemic PPI-rats showing expression of clusterin (brown) (compare (D) and (E) to control and hypergastrinemic PPI-rats in Fig 2A). (F-H) Number per oxyntic gland of (F) CLU-positive cells in IHC-stained sections (n = 5–8 rats per group), (G) dual CLU-positive/PGA5-positive cells (n = 4 rats per group), and (H) dual CLU-positive/PCNA-positive cells (n = 4 rats per group). Data presented as means ± SEM. *ANOVA with Tukey-adjusted *p* value < 0.05. Nuclei were counterstained with hematoxylin (blue). The basal zone (~100 μm from the gland bottom) is highlighted with a dotted line. Scale bars = 100 μm.

Previously, we have found that gastrin regulates expression of CLU *in vitro*, and PPI-induced hypergastrinemia increases the level of CLU in oxyntic mucosa of rats [13]. In the present study, ISH revealed that the change in CLU expression pattern was due to increased expression of *Clu* mRNA *de novo* (Fig 2A), and was not attributable only to increased secretion of CLU from neuroendocrine cells. In fact, there were significantly fewer neuroendocrine cells that highly expressed CLU in oxyntic mucosa of hypergastrinemic PPI-rats compared with controls (Fig 2B and 2C). On the contrary, in hypergastrinemic PPI-rats, the main cell type expressing high levels of CLU in oxyntic glands were basal chief cells, co-expressing either MIST1 or PGA5 (chief cell-markers) (Figs 2D and 3G) [36]. However, not all chief cells expressed high levels of CLU. The level of MIST1 in the nuclei and PGA5 and CLU in the cytoplasm seemed to vary between glands, resulting in three cell types: only chief cell marker-positive, both chief cell marker- and CLU-positive and only CLU-positive. Also, some basal CLU-positive chief cells were actively dividing (Figs 2D and 3H) and a few GSII-positive mucous neck cells co-expressed CLU in the lower neck of glands (S1B Fig).

To investigate whether those changes were dependent upon CCK2R signaling, rats were treated with PPI and/or the CCK2R antagonist netazepide. Elevated plasma concentrations of gastrin, and attenuated ECL cell and mucosal hyperplasia, confirmed efficient blockade of the CCK2R (Table 2 and Fig 3A) [34]. Levels of CLU protein in oxyntic mucosa lysates (Fig 3B) and plasma (Fig 3C) were increased by PPI-induced hypergastrinemia and decreased by blocking the CCK2R, reaching statistical significance in tissue lysates when comparing hypergastrinemic PPI-rats with or without CCK2R antagonist (Fig 3B).

**Table 2:** Biochemical and morphological characteristics of control rats and rats with PPI-induced hypergastrinemia, CCK2R antagonist treatment alone and PPI-induced hypergastrinemia in combination with CCK2R antagonist treatment.

	Control (n = 9)	PPI (n = 6)	CCK2R antagonist (n = 8)	PPI + CCK2R antagonist (n = 8)
**Gastrin start (pmol/L) (RIA)**	58 ± 7	61 ± 7	54 ± 7	48 ± 4
**Gastrin end (pmol/L) (RIA)**	26 ± 2	492 ± 31*	135 ± 22*	461 ± 31*
**Mucosal thickness (μm)**	471 ± 10	593 ± 28§	462 ± 13	487 ± 19
**Enterochromaffin-like (ECL) cell hyperplasia**	0/2	5/6	0/2	2/8

Plasma gastrin and mucosal thickness presented as mean ± standard error of mean (SEM).

*Bonferroni-adjusted p value < 0.01 when compared with control, analyzed with one-way ANOVA.

^§^Tukey-adjusted p value < 0.01 when comparing all groups, analyzed with one-way ANOVA

In most hypergastrinemic PPI-rats also receiving CCK2R antagonist, the main CLU expression pattern was still shifted to basal groups of chief cells (mRNA 5/5, protein 7/8) (Fig 3E). However, there was a trend towards fewer CLU-positive cells (Fig 3F), and less co-expression of CLU and chief cell markers (Fig 3G), and there were significantly fewer actively dividing CLU-positive cells (Fig 3H). In addition, despite also being hypoacidic and hypergastrinemic, the number of CLU-positive cells decreased (Fig 3D and 3F) after treatment with the CCK2R antagonist alone, compared with the controls. Overall, these findings show that expression of CLU in rat oxyntic mucosa is partly regulated by gastrin signaling through the CCK2R.

### Hypoacidity and hypergastrinemia causes upregulation of clusterin expression in the mucous neck cell-chief cell lineage

**Fig 4:**
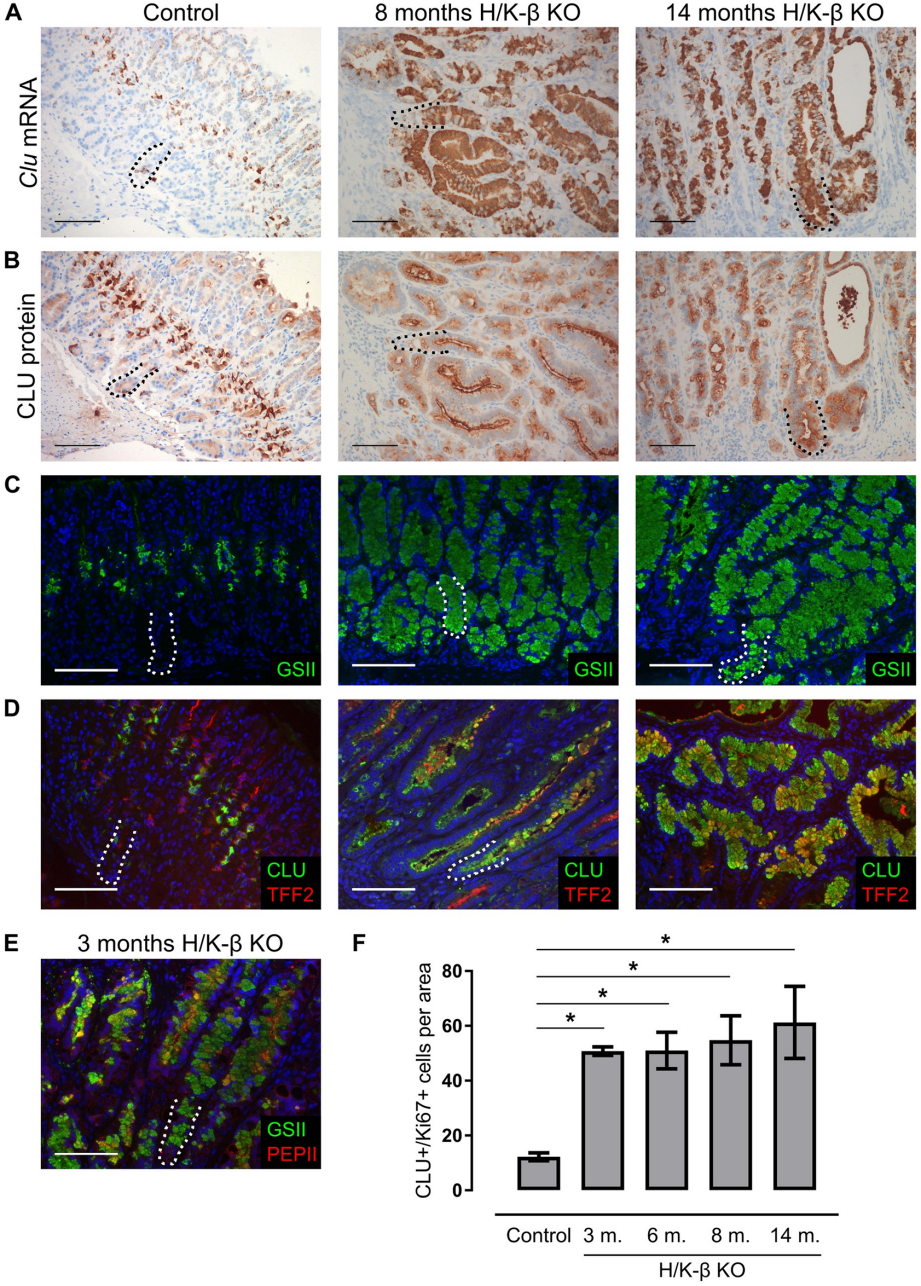
Clusterin and mucous neck cell markers are co-localized in oxyntic mucosa of H/K-β KO mice. (A, B) ISH and IHC showing overexpression of clusterin (brown) in oxyntic mucosa of H/K-β KO mice aged 8 months (n = 4) (middle column) and 14 months (n = 4) (right column) compared with wild-type control mice (n = 4) (left column). (C) Immunofluorescence staining showing increased GSII (green) expression in oxyntic mucosa of H/K-β KO mice aged 8 months and 14 months compared with wild-type control mice, particularly apparent in cells located in the basal half of glands. (D) Double immunofluorescence staining showing CLU (green) expression in TFF2-positive cells (red) in oxyntic mucosa from wild-type control mice and H/K-β KO mice aged 8 and 14 months. (E) Double immunofluorescence staining showing co-expression of GSII (green) and PEPII (red) in oxyntic mucosa from H/K-β KO mice aged 3 months. (F) Number of dual CLU-positive/Ki67-positive cells per area (0.44 mm^2^) of oxyntic mucosa (n = 4 mice per group). Data presented as means ± SEM. The basal zone (~100 μm from the gland bottom) is highlighted with a dotted line. *ANOVA with Tukey-adjusted *p* value < 0.05. Nuclei were counterstained with hematoxylin (blue) or DAPI (blue). Scale bars = 100 μm.

We wanted to test whether the pattern of CLU overexpression that we observed in rat oxyntic mucosa was a general feature of hypergastrinemic animal models. H/K-β KO mice are, like PPI-treated rats, hypo/anacidic and therefore hypergastrinemic [37, 38]. In H/K-β KO mice aged 3–14 months, the morphology of the oxyntic mucosa was fundamentally altered [26], and we found coincident, massive upregulation of CLU mRNA and protein (Fig 4A and 4B). Since CLU was co-localized with the mucous neck cell markers GSII (Fig 1D) and TFF2 (spasmolytic polypeptide) (Fig 4D) (in addition to neuroendocrine markers) in wild-type mice [17], we examined GSII and TFF2 also in H/K-β KO mice. In distinct areas of their hyperplastic mucosa, there were mucous cells expressing high levels of GSII (Fig 4C), TFF2 (Fig 4D), and mixed periodic acid Schiff and Alcian blue positivity (S2A Fig), but no mucin 2 (S2B Fig). Localized basally in several glands, there were CLU-positive cells co-expressing either TFF2 (Fig 4D) or GSII (S2C Fig), (hybrid) cells co-expressing GSII and the chief cell marker pepsinogen II (Fig 4E), and significantly more numerous proliferating CLU-positive cells than in wild-type controls (Fig 4F and S2D Fig). Interestingly, SPEM is characterized by expression of TFF2 or GSII in chief cell marker-positive (hybrid) cells in the base of oxyntic glands, often with increased proliferation [6, 39], and those changes, together with overexpression of CLU, can be used as markers for SPEM [17, 23].

**Fig 5:**
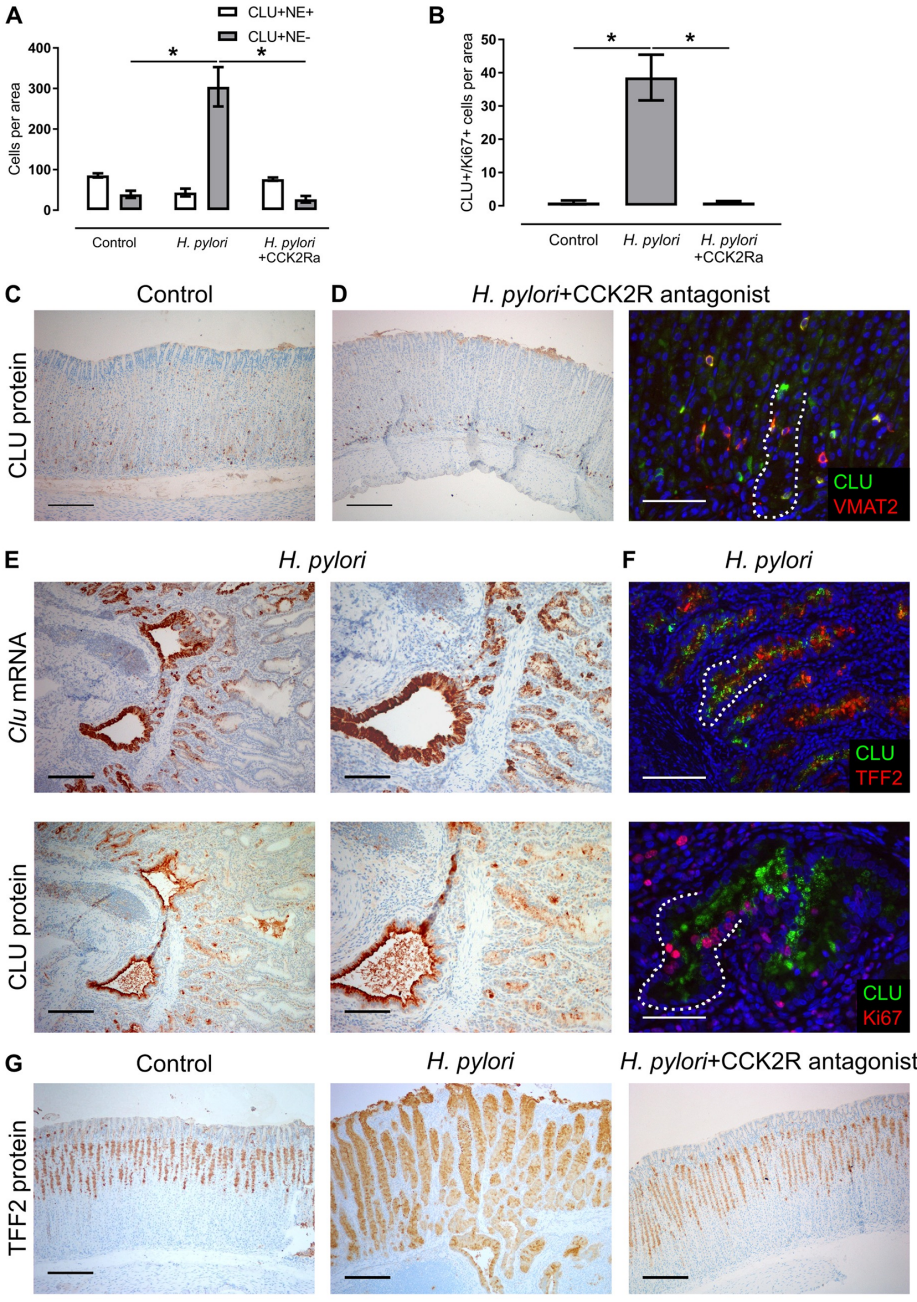
Clusterin and TFF2 expression in oxyntic mucosa of Mongolian gerbils increase after *H*. *pylori* infection and normalize when antagonizing the CCK2R. (A, B) Number per oxyntic area (0.44 mm^2^) of (A) CLU-positive/VMAT2(neuroendocrine (NE))-positive cells and CLU-positive/VMAT2(NE)-negative cells and (B) dual CLU-positive/Ki67-positive cells from uninfected control (n = 4), *H*. *pylori*-infected (*H*. *pylori*) (n = 7), and CCK2R-antagonized *H*. *pylori*-infected gerbils (*H*. *pylori*+CCK2Ra) (n = 4). (C, D) Oxyntic expression of CLU (brown) and dual CLU-positive (green)/VMAT2-positive (red) ECL cells in (C) uninfected control (n = 4) and (D) CCK2R-antagonized *H*. *pylori*-infected gerbils (n = 4). (E) ISH and IHC showing expression of clusterin in oxyntic mucosa of *H*. *pylori*-infected gerbils (n = 7). (F) Double immunofluorescence staining showing co-expression of CLU (green) and TFF2 (red, upper figure) and CLU (green) and Ki67 (red, lower figure) in oxyntic mucosa of *H*. *pylori*-infected gerbils. (G) IHC showing TFF2 expression (brown) in oxyntic mucosa of uninfected controls and *H*. *pylori*-infected gerbils untreated and treated with a CCK2R antagonist. Data presented as means ± SEM. *ANOVA with Tukey-adjusted *p* value < 0.05. Nuclei were counterstained with hematoxylin (blue) or DAPI (blue). The basal zone (~100 μm from the gland bottom) is highlighted with a dotted line. Scale bars = (C, D left column, E left column, G) 200 μm; (E right column, F upper figure) 100 μm; (D right column, F lower figure) 50 μm.

In hypergastrinemic *H*. *pylori*-infected Mongolian gerbils, the CLU expression pattern also shifted compared with control (Fig 5A and 5C). CLU was highly expressed in the basal half of several metaplastic and invasive glands (Fig 5E), particularly co-expressed with TFF2 and Ki67 (Fig 5B and 5F), similar to previous findings in human and rodent SPEM [17, 23]. In contrast, CLU expression in oxyntic mucosa of CCK2R-antagonized *H*. *pylori*-infected gerbils was restricted to non-proliferative single cells, including ECL cells (Fig 5A, 5B and 5D), and there were no visible signs of SPEM (Fig 5G), as the mucosa in general was unchanged from uninfected controls [27]. Taken together, these results indicate that, in different animal models with hypergastrinemia due to diminished parietal cell proton pump function or *H*. *pylori*-infection, CLU is overexpressed in basal groups of (metaplastic) cells from the mucous neck cell-chief cell lineage, and that overexpression can be inhibited partially by antagonizing the CCK2R.

### Human gastric adenocarcinoma cells express and secrete clusterin

**Fig 6:**
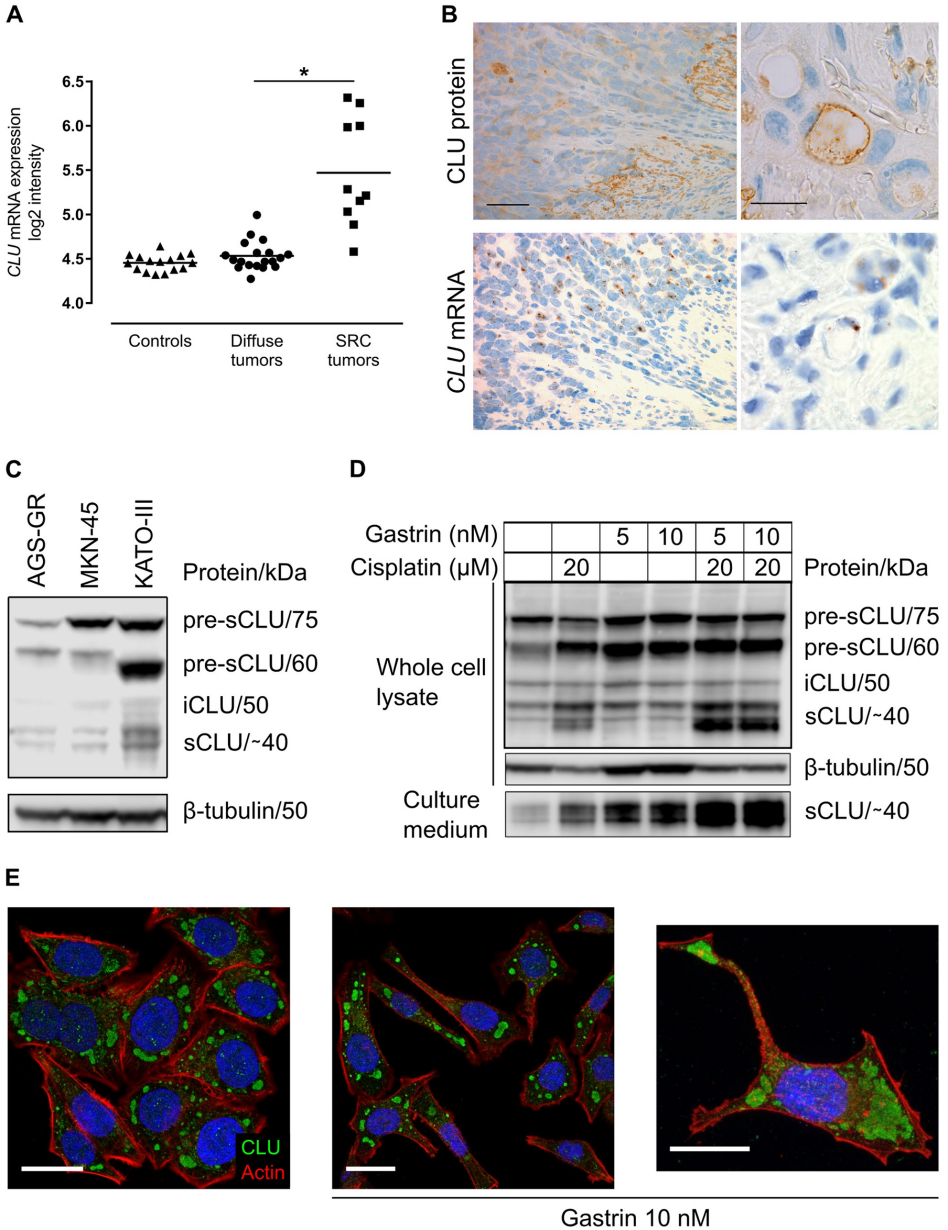
Human gastric adenocarcinoma cells express and secrete clusterin in response to gastrin and cisplatin. (A) In-house gene expression dataset (ILMN_1815184 Illumina HumanHT-12) showing higher expression of *CLU* mRNA in diffuse tumors containing signet ring cells (SRC tumors) compared with tumors without SRCs (diffuse tumors). Each dot represents individual mRNA expression levels (log2 intensities) and the black lines mark mean. *corrected *p* value < 0.0001. (B) IHC of CLU protein (brown) and ISH of *CLU* mRNA (brown) showing localization in tumor cells in human diffuse SRC tumors. (C) Western blot showing different isoforms of CLU expressed in the gastric cancer cell lines AGS-GR, MKN-45 and KATO-III. (D) Western blot showing that gastrin and/or cisplatin for 48 hours stimulated increased expression and secretion (lower panel) of CLU in AGS-GR cells. Similar results were found after 24 hours (S3 Fig). The image is representative of 3 independent experiments. β-tubulin was used as loading control. pre-sCLU = precursor of secretory CLU; iCLU = intracellular CLU; sCLU = secretory CLU. (E) Immunocytochemical staining of AGS-GR cells showing cytoplasmic vesicular expression of CLU (green) in unstimulated cells or cells treated with gastrin for 24 hours. Actin is stained with Rhodamine Phalloidin (red) and nuclear DNA with DAPI (blue). Scale bars = (B left column) 50 μm; (B right column, E) 20 μm.

Given our findings in normal and premalignant rodent mucosa, and since CLU has been found by immunohistochemistry to be expressed in human SPEM and gastric cancer [17, 19, 21], we did further examinations in human gastric cancer material. Our in-house gene expression dataset (96 samples) showed no difference in *CLU* mRNA expression in adenocarcinomas (of both intestinal and diffuse type) compared with normal or adjacent non-tumor mucosa. However, further sub-analyses revealed significantly higher *CLU* expression in diffuse tumors containing SRCs compared with diffuse tumors without SRCs (fold change 1.935) (Fig 6A). Likewise, Oncomine analyses showed mainly unchanged *CLU* mRNA expression in all types of gastric adenocarcinomas compared with normal mucosa (5 sets, 478 samples), and significantly increased expression mainly in diffuse tumors (without specification of SRCs) [40–42] *versus* intestinal [42] and mixed [43] tumors when comparing histological subtypes (5 sets, 534 samples). In SRCs tumors, we observed by immunohistochemistry apparently stronger expression of CLU protein in extracellular matrix than in tumor cells (Fig 6B). However, ISH revealed that mainly tumor cells expressed *CLU* mRNA, indicating that CLU protein was secreted from tumor cells into extracellular matrix. Both CLU protein and mRNA expression were seen in some, but not all, SRCs (Fig 6B).

Next, we performed western blot analysis to show expression of CLU in three human gastric cancer cell lines (Fig 6C). In AGS-GR cells, CLU was localized to seemingly small vesicles in the perinuclear area and in plasma membrane extensions (Fig 6E). Furthermore, expression and secretion of CLU from AGS-GR cells increased in response to 24 or 48 hours’ treatment with the known stress-inducer cisplatin, and increased even more after treatment with the hormone gastrin or the two in combination (S3 Fig and Fig 6D). Combined, our results show that *CLU* is increased in diffuse SRC adenocarcinomas, and that gastric cancer cells display signal-induced production and secretion of CLU.

### Secretory clusterin can increase survival of gastric cancer cells

**Fig 7:**
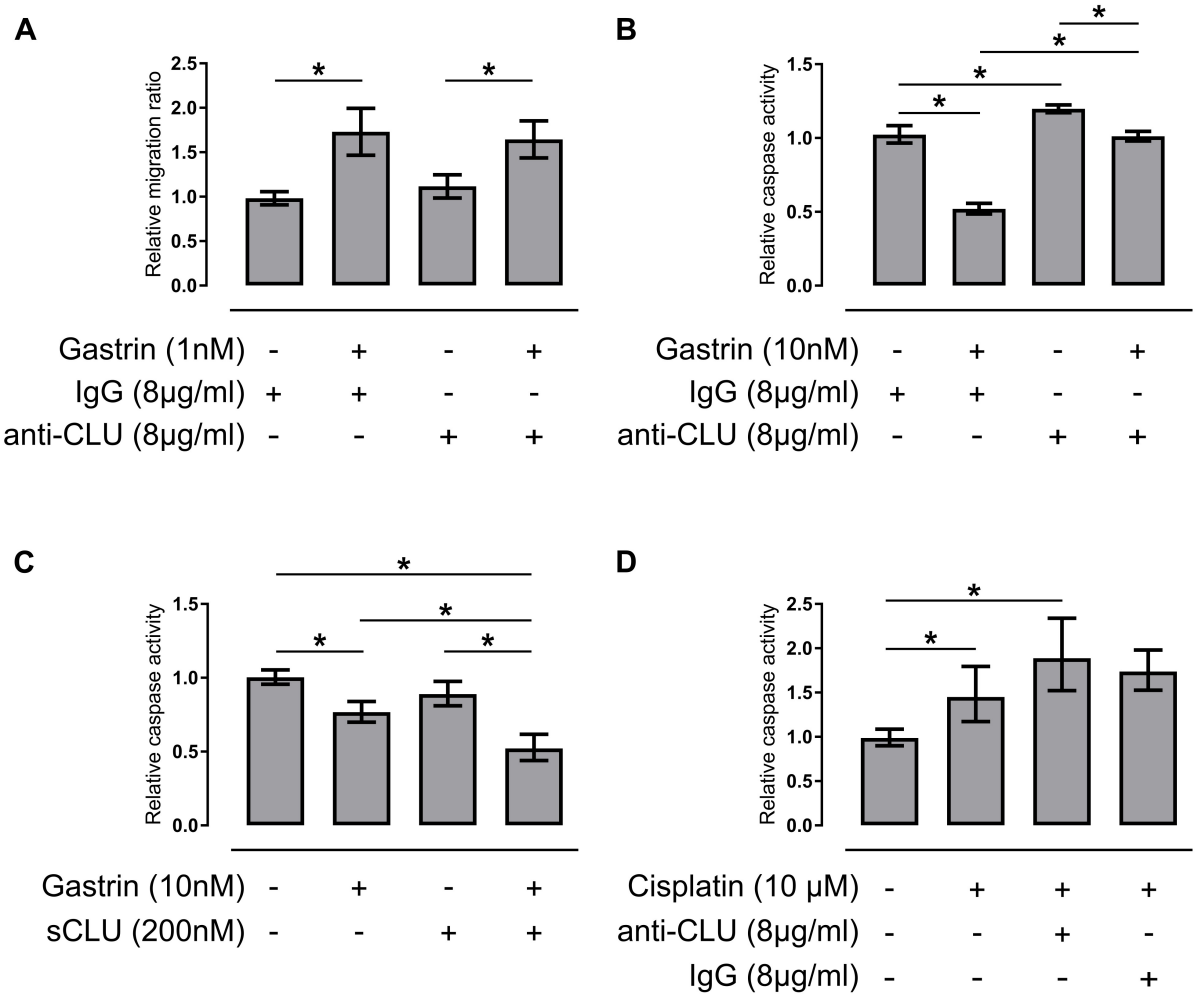
Secretory clusterin promotes survival of gastric cancer cells after starvation- and chemotherapy-induced stress. (A) Quantification of basal and gastrin-induced migration of AGS-GR cells after 18 hours in the presence of either non-immunized IgG (8 μg/ml) or anti-clusterin (anti-CLU) (8 μg/ml). Relative migration ratio was estimated using data from 5 independent experiments (2–4 technical replicates in each experiment), normalizing the data to the median cell index of untreated cells with non-immunized IgG in each independent experiment. (B-D) Apoptosis was induced by serum-starvation for 48 hours and caspase 3/7 activity measured in: (B) AGS-GR cells grown in the presence of either non-immunized IgG (8 μg/ml) or anti-CLU (8 μg/ml) with or without gastrin (10 nM). Relative caspase activity was estimated using data from 2–5 independent experiments (2–6 technical replicates in each experiment), normalizing the data to the median intensity of cells with only non-immunized IgG present in each independent experiment. (C, D) AGS-GR cells grown in (C) the absence or presence of gastrin (10 nM), sCLU (200 nM), or both; (D) the absence or presence of cisplatin (10 μM) combined with either anti-CLU (8 μg/ml) or non-immunized IgG (8 μg/ml). Relative caspase activity was estimated using data from 3–4 independent experiments (6 technical replicates in each experiment), normalizing the data to the median intensity of untreated cells in each independent experiment. Data is presented as mean with error bars representing 95% confidence intervals. *Students t-test with Bonferroni-adjusted *p* value < 0.05.

Both gastrin and sCLU are known to stimulate cell migration/invasion and inhibit apoptosis [15, 16, 33, 44–46]. Since we found high levels of CLU in areas of invasive growth in *H*. *pylori*-infected Mongolian gerbils (Fig 5E) and gastrin induced secretion of CLU in AGS-GR cells (Fig 6D), we hypothesized that sCLU could play a part in gastrin-induced migration. As expected, gastrin increased cell migration by ~70% (Fig 7A) [45] in AGS-GR cells and not AGSwt cells (S4B and S4C Fig). However, adding neutralizing anti-CLU antibodies (C-18) [33] (Fig 7A) or recombinant sCLU (S4D Fig) did not affect basal or gastrin-induced migration.

Gastrin showed a reproducible anti-apoptotic effect on 48 hours’ serum starvation-induced apoptosis (Fig 7B and 7C) [45]. Neutralization of sCLU with anti-CLU antibodies (C-18) [33] had a significant pro-apoptotic effect, and partly reversed the anti-apoptotic effect of gastrin (Fig 7B). Recombinant sCLU and gastrin and sCLU together yielded a strong anti-apoptotic effect (Fig 7C and S7 Fig). AGS cells have low sensitivity to the cytostatic cisplatin (IC50 value 13.6 μM [47]), normally used in perioperative treatment of gastric cancer [48]. Neutralizing sCLU during cisplatin treatment of AGS-GR cells had a pro-apoptotic effect compared with cisplatin alone, although the effect was not significant by post hoc analysis (*p* value = 0.10) (Fig 7D). These findings suggest that secretion of CLU can promote increased survival of gastric cancer cells after gastrin stimulation and prolonged starvation- or chemotherapy-induced stress.

## Discussion

In this study, we investigated the expression and regulation of CLU in oxyntic mucosa of hypergastrinemic rodent models and humans, and elucidated the function of sCLU in gastric cancer cells during stress. In normal rat, mouse and gerbil oxyntic mucosa, we identified the prominent single cells that highly expressed CLU as ECL cells and A-like cells. We found a similar pattern in human oxyntic mucosa, which contrasts with a previous report describing expression of CLU only in the isthmus [17]. Nevertheless, the picture in that report seems to show basal CLU-positive single cells as well. Also, CLU is expressed in neuroendocrine cells in colon and pancreas [49, 50], and is overexpressed in some gastric and pancreatic neuroendocrine tumors [50, 51]. The CLU expression pattern in oxyntic mucosa of wild-type mice was different from other rodents and humans, with prominent expression in both neuroendocrine cells and mucous neck cells. The significance of this difference is not known. However, it is intriguing that CLU consistently is present in cells from the neuroendocrine or mucous neck cell-chief cell lineages, and not in parietal cells, indicating that CLU is differentially expressed by peptide- and/or mucus-secretory cell types.

In rats, after PPI-induced acid inhibition with subsequent hypergastrinemia, a few neck cells and several basal groups of chief cells massively increased their expression of CLU. Some gland bases with highly CLU-positive cells expressed chief cell markers (MIST1 and PGA5) at low or undetectable levels, distinguishing them from surrounding chief cells and suggesting that they might represent a subtype. In addition, some of the CLU-positive chief cells appeared to be actively dividing. Downregulation of MIST1, loss of PGA5, increased expression of CLU, and emergence of proliferating cells at the base of oxyntic glands are associated with chief cell transdifferentiation and development of SPEM, which are normally induced by oxyntic atrophy (parietal cell loss) [17, 36, 39]. Pharmacological inhibition (PPI) of parietal cell proton pump function and subsequent hypergastrinemia could mimic some features of parietal cell loss, and one might speculate that the proliferating CLU-positive chief cells represent a variant of chief cell transdifferentiation. Importantly, we did not observe definite SPEM with (hybrid) cells co-expressing chief and mucous neck cell markers in the base of oxyntic glands.

The changes in oxyntic CLU expression were partly reversed in CCK2R-antagonized PPI-rats and were absent or downregulated in rats given CCK2R antagonist alone. The CCK2R antagonist alone also causes hypoacidity and hypergastrinemia, but less so than PPI, and the parietal cell proton pump is not directly affected. Thus, our findings show that blocking CCK2R signaling affects oxyntic CLU expression *in vivo* and that, in hypergastrinemic PPI-rats, CLU expression is upregulated and shifted to chief cells by both gastrin-dependent and gastrin-independent pathways. In cells expressing the CCK2R, gastrin can induce expression of *Clu* through the AP-1 transcription factor complex; as the clusterin promoter contains an AP-1 responsive element and gastrin regulates expression of the AP-1 complex members *c-fos* and *junB* [13, 52]. In CCK2R-negative cells, like chief cells are normally reckoned to be [53], the gastrin-dependent pathway for regulation of CLU expression is most likely mediated indirectly through factors released from ECL cells or parietal cells [54, 55], such as epidermal growth factor receptor (EGFR) ligands, which indeed can induce expression of *Clu* [56]. Independent of gastrin, it seems likely that hypoacidity per se, diminished proton pump function, alteration of CLU-repressive or -inducing signals from PPI-targeted parietal cells, abnormal activity of chief cells (altered pepsinogen cleavage), bacterial overgrowth, and more, could also influence CLU expression in oxyntic mucosa, as several of these factors, in addition to hypergastrinemia, are present in the rodent models where CLU is overexpressed.

H/K-β KO mice are a non-pharmacological model of potent acid inhibition and hypergastrinemia, with abnormal development of oxyntic glands [26, 37, 38, 57]. In this study, we thoroughly confirm that CLU is highly upregulated in oxyntic mucosa of H/K-β KO mice of different ages [58]. CLU-positive cells in H/K-β KO mice proliferated and co-localized with mucous neck cell markers located in the base of elongated mucinous glands, next to (hybrid)cells co-expressing GSII and pepsinogen II, a pattern typical of SPEM [6, 7]. Intriguingly, in these mice, removal of a functional subunit of the proton pump, rather than loss of parietal cells per se, seems to trigger emergence of those oxyntic mucosal changes. Thus, our results suggest that H/K-β KO mice develop CLU-positive SPEM, in addition to the severe mucosal disruption previously described [26, 37, 38], which supports the notion that the H/K-β KO mouse is a novel spontaneous SPEM model [57].

Another SPEM model is Mongolian gerbils infected with *H*. *pylori* [8, 23]. Within that model, we identified the expression pattern of *Clu* mRNA and confirmed that these animals developed CLU-positive SPEM. Sørdal et al. [27] reported that the CCK2R antagonist netazepide prevented development of gastritis and subsequent pathological changes in oxyntic mucosa, after *H*. *pylori* infection. Similarly, in oxyntic mucosa of CCK2R-antagonized *H*. *pylori*-infected gerbils, we found no areas of SPEM and the CLU expression pattern remained identical to non-infected controls, indicating that gastrin signaling through the CCK2R plays an important role in development of *H*. *pylori*-induced SPEM in this model.

Overall, in three animal models with hypergastrinemia due to diminished parietal cell proton pump function or *H*. *pylori*-infection, CLU was overexpressed in basal groups of proliferative (and metaplastic) cells in the mucous neck cell-chief cell lineage, and that overexpression could be partially inhibited by antagonizing the CCK2R. Medically- or *H*. *pylori*-induced oxyntic atrophy induces SPEM [6, 39]. However, a recent report indicates that targeted apoptosis of parietal cells is insufficient to induce metaplasia [59], thus, the exact mechanisms are unknown. A common factor is affection of parietal cell proton pump function and absence of gastric acid secretion, which leads to hypergastrinemia. One study shows that gastrin protects against development of SPEM [60], while others report that gastrin promotes metaplastic transformation [61, 62]. Indeed, H+/K+-ATPase β-subunit/gastrin double-KO mice did not develop SPEM-like “mucus-rich” cell hyperplasia [57]. That, together with our findings, indicates that, although gastrin is not essential for the development of SPEM, it might influence the metaplastic cascade, possibly through upregulating CLU expression in hypoacidic oxyntic glands. Upregulation of CLU could make metaplastic cells more resistant to harmful stimuli, such as oxidative stress due to chronic inflammation [18], and oxidative stress may play a central role in gastric tumorigenesis [63].

CLU is dysregulated in multiple cancers, including gastric ones, in which overexpression seems to correlate with cancer progression [17, 19, 21]. The suggested U-shaped correlation pattern probably explains why overexpression could be hidden in large-scale analysis of heterogeneous samples. Interestingly, we identified a difference in *CLU* expression between histological subtypes of gastric adenocarcinomas, with higher *CLU* expression in diffuse tumors containing SRCs compared with diffuse tumors without SRCs. The diffuse SRC tumors represent a subtype with possible distinct clinicopathologic characteristics and prognosis than other diffuse cancers [64–66]. Our findings suggest that CLU could be one of several mediators contributing to these characteristic features. Whether there is a link between the expression patterns of CLU in gastric normal mucosa and metaplasia, and in diffuse gastric cancer with SRCs, is not known. The diffuse SRC tumors are not typically thought to develop on the background of metaplastic changes. Still, SPEM (and clusterin expression) and intestinal metaplasia have been found in adjacent gastric mucosa of diffuse tumors [17, 19, 67, 68] and another SPEM-marker (WAP four-disulfide core domain protein 2 (WFDC2)) is also strongly expressed in diffuse SRC tumors [69]. *CLU* was mainly localized to tumor cells but the protein apparently was readily secreted. Furthermore, gastric cancer cell lines expressed and secreted CLU upon stimulation with stressors like gastrin and cisplatin. Both gastrin and CLU might promote cell migration/invasion [16, 33, 44, 46], and, even though sCLU did not affect migration of AGS-GR cells, our findings do not exclude a pro-migratory role of CLU in other gastric cancer cell lines or *in vivo*.

Both gastrin and sCLU alone promoted an anti-apoptotic effect on gastric cancer cells, and it is noteworthy that gastrin and sCLU in combination enhanced survival even further, particularly on early apoptosis signaling. We confirmed that sCLU is directly involved in the anti-apoptotic effect of gastrin [13], and a possible indirect involvement could be mediated by CLU’s activation of autophagy [45, 70, 71]. sCLU functions as an extracellular molecular chaperone, enabling it to bind a wide array of peptides, including misfolded or denatured proteins, cellular debris, lipids and other potentially harmful molecules; thereby preventing that they form insoluble aggregates, bind to receptors, or impede damage on neighboring cells [14, 15, 18]. This scavenging function of sCLU is cytoprotective, and is highly relevant in stressed cells, where damaging agents accumulate and need to be cleared away to avoid cell death [15, 18].

CLU might also reach the cytoplasm, either by alternative transcription or splicing, failed translocation, or retrotranslocation from the endoplasmic reticulum (ER)-Golgi pathway [15]. Intracellularly, CLU has been found to bind and inactivate apoptosis regulator BAX (BAX), thereby altering the ratio between pro-apoptotic BAX and anti-apoptotic B-Cell CLL/Lymphoma 2 proteins towards a pro-survival level [72, 73]. Interestingly, we observed an increase in putative intracellular forms of CLU (60 and 75 kDa precursors of sCLU) in gastric cancer cells in response to gastrin and/or cisplatin, but without further comprehensive analyses, we do not know whether these peptides are exclusively located in the ER-Golgi-pathway, waiting to be secreted, or also locate to the cytoplasm, possibly interacting with mitochondrial and apoptosis-related proteins. Indeed, immunocytochemistry of gastric cancer cells showed that CLU was mainly located in the perinuclear area and towards the cell membrane, suggestive of secretory pathway localization. Additionally, sCLU decreased the cytotoxicity of cisplatin on gastric cancer cells, similarly as gastrin [45], and both might be involved in treatment resistance. Taken together, these results suggest that gastrin and sCLU can make gastric cells more resistant to stress-induced cell death.

In summary, we have shown that CLU is highly expressed in oxyntic mucosa of hypergastrinemic rodent models, particularly in glands containing putative metaplastic cells from the mucous neck cell-chief cell lineage, and that CLU expression is partly regulated by gastrin *in vivo*. Furthermore, cisplatin and gastrin made gastric cancer cells express and secrete CLU, leading to increased survival and possibly treatment resistance. Overall, our results indicate that CLU could be involved in gastrin-induced pro-survival signaling and remodeling of the oxyntic mucosa; and might therefore influence both gastric homeostasis and cancer risk.

## Supporting information

S1 Fig Clusterin expression in a few mucous neck cells, but no parietal cells. (A) IHC of oxyntic/corpus mucosa from control rat, Mongolian gerbil and mouse showing no expression of CLU (brown) in parietal cells (arrows). (B) Double immunofluorescence staining of oxyntic mucosa from hypergastrinemic PPI-rats showing CLU (red) expression in a few mucous neck cells (GSII-positive (green)) in the lower neck region of oxyntic glands. Nuclei were counterstained with hematoxylin (blue) or DAPI (blue). The basal zone (~100 μm from the gland bottom) is highlighted with a dotted line. Scale bars (A middle and right column) = 100 μm; (A left column, B) 50 μm.
(TIF)

S2 Fig Mucous and proliferation marker staining of oxyntic mucosa from H/K-β KO mice. (A) Periodic acid Schiff and Alcian blue staining of oxyntic mucosa from wild-type control and H/K-β KO mice aged 6 months. (B) IHC staining of oxyntic mucosa from H/K-β KO mice aged 8 months showing no mucin 2 expression in the gastric epithelial cells. There is some positivity in scattered single cells in the underlying stroma. (C) Double immunofluorescence staining of oxyntic mucosa from H/K-β KO mice aged 6 months showing CLU (red) expression in GSII-positive cells (green). (D) Double immunofluorescence staining of oxyntic mucosa from wild-type control mice and H/K-β KO mice aged 14 months showing co-expression of CLU (green) and the proliferation marker Ki67 (red). Nuclei were counterstained with hematoxylin (blue) or DAPI (blue). The basal zone (~100 μm from the gland bottom) is highlighted with a dotted line. Scale bars = 100 μm.
(TIF)

S3 Fig Expression and secretion of clusterin after treatment with gastrin and/or cisplatin for 24 hours. (A) Western blot showing that gastrin and/or cisplatin for 24 hours stimulated increased expression of CLU in AGS-GR cells. (B) β-tubulin was used as loading control. Left image is shown with low contrast. Right image is shown with high contrast, in order to visualize the molecular size marker. (C) Western blot of the pertaining culture medium showing that gastrin and/or cisplatin for 24 hours stimulated increased secretion of sCLU from AGS-GR cells. (D) Due to a technical issue with the lane for gastrin 10 nM in (C), resulting in weak signals, we demonstrate an additional gel blot, from an independent experiment, showing that gastrin and/or cisplatin for 24 or 48 hours stimulated increased secretion of sCLU from AGS-GR cells. pre-sCLU = precursor of secretory CLU; iCLU = intracellular CLU; sCLU = secretory CLU.
(TIF)

S4 Fig Influence of gastrin and recombinant secretory clusterin on migration of human gastric cancer cells. (A) Western blot of culture medium showing that gastrin or cisplatin for 48 hours stimulated increased secretion of sCLU in AGS-GR and not AGS wild type (AGSwt) cells. sCLU = secretory CLU. (B) Quantification of migration of AGS-GR and AGSwt cells after 18 hours in the absence or presence of gastrin 1 nM. Relative migration ratio was estimated using data from 5 independent experiments (2–7 technical replicates in each experiment) with AGS-GR and 2 independent experiments (3–5 technical replicates in each experiment) with AGSwt. (C) Quantification of migration, as number of pixels migrated, of AGS-GR cells in a scratch assay after 15 hours in the absence or presence of gastrin 1 nM or 10 nM. Data from 3 independent experiments. (D) Quantification of migration of AGS-GR cells after 18 hours in the presence of recombinant sCLU at 50 or 200 nM. Relative migration ratio was estimated using data from 2 independent experiments (2–3 technical replicates in each experiment). Data was normalized to the median cell index of untreated cells in each independent experiment. Data is presented as means with error bars representing 95% confidence intervals. *Students t-test with Bonferroni-adjusted *p* value < 0.05.
(TIF)

S5 Fig Uncropped gel blots for Fig 6C. Western blot showing different isoforms of CLU expressed in the gastric cancer cell lines AGS-GR, MKN-45 and KATO-III, cultured with and without fetal calf serum (FCS). β-tubulin was used as loading control. pre-sCLU = precursor of secretory CLU; iCLU = intracellular CLU; sCLU = secretory CLU.
(TIF)

S6 Fig Uncropped gel blots for Fig 6D. (A) Western blot showing that gastrin and/or cisplatin for 48 hours stimulated increased expression of CLU in AGS-GR cells. (B) β-tubulin was used as loading control. Left image is shown with low contrast. Right image is shown with high contrast, in order to visualize the molecular size marker. (C) Western blot of the pertaining culture medium showing that gastrin and/or cisplatin for 48 hours stimulated increased secretion of sCLU from AGS-GR cells. pre-sCLU = precursor of secretory CLU; iCLU = intracellular CLU; sCLU = secretory CLU.
(TIF)

S7 Fig Secretory clusterin promotes survival of gastric cancer cells after starvation-induced stress. Apoptosis was induced by serum-starvation for 72 hours and TUNEL staining quantified as absorbance at 450 nm in AGS-GR cells grown in the absence or presence of gastrin (10 nM), sCLU (200 nM), or both. A positive control with nuclease treatment of cells showed absorbance 3.67, and a negative control with omission of the reaction enzyme showed absorbance 0.27. Data is presented as mean of 6 technical replicates with error bars representing 95% confidence intervals. Results are representative for two independent experiments (6 technical replicates in each experiment). *ANOVA with Tukey-adjusted *p* value < 0.05.
(TIF)

S1 Table Targeted MS—Transition results. (XLSX)

## References

[1] Choi E, Roland JT, Barlow BJ, O'Neal R, Rich AE, Nam KT, et al Cell lineage distribution atlas of the human stomach reveals heterogeneous gland populations in the gastric antrum. Gut. 2014;63(11):1711–20. doi: 10.1136/gutjnl-2013-305964 24488499 10.1136/gutjnl-2013-305964 PMC4117823

[2] Karam SM, Straiton T, Hassan WM, Leblond CP. Defining epithelial cell progenitors in the human oxyntic mucosa. Stem Cells. 2003;21(3):322–36. doi: 10.1634/stemcells.21-3-322 12743327 10.1634/stemcells.21-3-322

[3] Vange P, Bruland T, Beisvag V, Erlandsen SE, Flatberg A, Doseth B, et al Genome-wide analysis of the oxyntic proliferative isthmus zone reveals ASPM as a possible gastric stem/progenitor cell marker over-expressed in cancer. The Journal of pathology. 2015;237(4):447–59. doi: 10.1002/path.4591 26178168 10.1002/path.4591 PMC5049620

[4] Correa P, Haenszel W, Cuello C, Tannenbaum S, Archer M. A model for gastric cancer epidemiology. Lancet. 1975;2(7924):58–60. 49653 10.1016/s0140-6736(75)90498-5

[5] Correa P, Piazuelo MB. The gastric precancerous cascade. Journal of digestive diseases. 2012;13(1):2–9. doi: 10.1111/j.1751-2980.2011.00550.x 22188910 10.1111/j.1751-2980.2011.00550.x PMC3404600

[6] Goldenring JR, Nam KT, Mills JC. The origin of pre-neoplastic metaplasia in the stomach: chief cells emerge from the Mist. Experimental cell research. 2011;317(19):2759–64. doi: 10.1016/j.yexcr.2011.08.017 21907708 10.1016/j.yexcr.2011.08.017 PMC3210373

[7] Goldenring JR, Nam KT, Wang TC, Mills JC, Wright NA. Spasmolytic polypeptide-expressing metaplasia and intestinal metaplasia: time for reevaluation of metaplasias and the origins of gastric cancer. Gastroenterology. 2010;138(7):2207–10, 10 e1 doi: 10.1053/j.gastro.2010.04.023 20450866 10.1053/j.gastro.2010.04.023 PMC3769643

[8] Yoshizawa N, Takenaka Y, Yamaguchi H, Tetsuya T, Tanaka H, Tatematsu M, et al Emergence of spasmolytic polypeptide-expressing metaplasia in Mongolian gerbils infected with Helicobacter pylori. Laboratory investigation; a journal of technical methods and pathology. 2007;87(12):1265–76. doi: 10.1038/labinvest.3700682 18004396 10.1038/labinvest.3700682

[9] Burkitt MD, Varro A, Pritchard DM. Importance of gastrin in the pathogenesis and treatment of gastric tumors. World journal of gastroenterology: WJG. 2009;15(1):1–16. 19115463 10.3748/wjg.15.1 PMC2653300

[10] Waldum HL, Kleveland PM, Brenna E, Bakke I, Qvigstad G, Martinsen TC, et al Interactions between gastric acid secretagogues and the localization of the gastrin receptor. Scandinavian journal of gastroenterology. 2009;44(4):390–3. doi: 10.1080/00365520802624219 19089789 10.1080/00365520802624219

[11] Watson SA, Grabowska AM, El-Zaatari M, Takhar A. Gastrin—active participant or bystander in gastric carcinogenesis? Nature reviews Cancer. 2006;6(12):936–46. doi: 10.1038/nrc2014 17128210 10.1038/nrc2014

[12] Fossmark R, Qvigstad G, Martinsen TC, Hauso O, Waldum HL. Animal models to study the role of long-term hypergastrinemia in gastric carcinogenesis. Journal of biomedicine & biotechnology. 2011;2011:975479. 21127707 10.1155/2011/975479 PMC2992820

[13] Fjeldbo CS, Bakke I, Erlandsen SE, Holmseth J, Laegreid A, Sandvik AK, et al Gastrin upregulates the prosurvival factor secretory clusterin in adenocarcinoma cells and in oxyntic mucosa of hypergastrinemic rats. American journal of physiology Gastrointestinal and liver physiology. 2012;302(1):G21–33. doi: 10.1152/ajpgi.00197.2011 21995960 10.1152/ajpgi.00197.2011

[14] Rizzi F, Bettuzzi S. The clusterin paradigm in prostate and breast carcinogenesis. Endocrine-related cancer. 2010;17(1):R1–17. doi: 10.1677/ERC-09-0140 19903745 10.1677/ERC-09-0140

[15] Rohne P, Prochnow H, Koch-Brandt C. The CLU-files: disentanglement of a mystery. Biomol Concepts. 2016;7(1):1–15. doi: 10.1515/bmc-2015-0026 26673020 10.1515/bmc-2015-0026

[16] Zoubeidi A, Gleave M. Small heat shock proteins in cancer therapy and prognosis. The international journal of biochemistry & cell biology. 2012;44(10):1646–56. 22571949 10.1016/j.biocel.2012.04.010

[17] Weis VG, Sousa JF, LaFleur BJ, Nam KT, Weis JA, Finke PE, et al Heterogeneity in mouse spasmolytic polypeptide-expressing metaplasia lineages identifies markers of metaplastic progression. Gut. 2013;62(9):1270–9. doi: 10.1136/gutjnl-2012-302401 22773549 10.1136/gutjnl-2012-302401 PMC3762676

[18] Trougakos IP. The molecular chaperone apolipoprotein J/clusterin as a sensor of oxidative stress: implications in therapeutic approaches—a mini-review. Gerontology. 2013;59(6):514–23. doi: 10.1159/000351207 23689375 10.1159/000351207

[19] Bi J, Guo A, Lai R, Li B, Zhong J, Wu H, et al Overexpression of clusterin correlates with tumor progression, metastasis in gastric cancer: a study on tissue microarrays. Neoplasma. 2010;57(3):191–7. 20353268 10.4149/neo_2010_03_191

[20] Humphries JM, Penno MA, Weiland F, Klingler-Hoffmann M, Zuber A, Boussioutas A, et al Identification and validation of novel candidate protein biomarkers for the detection of human gastric cancer. Biochimica et biophysica acta. 2014;1844(5):1051–8. doi: 10.1016/j.bbapap.2014.01.018 24512919 10.1016/j.bbapap.2014.01.018

[21] Liu W, Liu B, Cai Q, Li J, Chen X, Zhu Z. Proteomic identification of serum biomarkers for gastric cancer using multi-dimensional liquid chromatography and 2D differential gel electrophoresis. Clinica chimica acta; international journal of clinical chemistry. 2012;413(13–14):1098–106. doi: 10.1016/j.cca.2012.03.003 22446497 10.1016/j.cca.2012.03.003

[22] Penno MA, Klingler-Hoffmann M, Brazzatti JA, Boussioutas A, Putoczki T, Ernst M, et al 2D-DIGE analysis of sera from transgenic mouse models reveals novel candidate protein biomarkers for human gastric cancer. Journal of proteomics. 2012;77:40–58. doi: 10.1016/j.jprot.2012.07.002 22789672 10.1016/j.jprot.2012.07.002

[23] Shimizu T, Choi E, Petersen CP, Noto JM, Romero-Gallo J, Piazuelo MB, et al Characterization of progressive metaplasia in the gastric corpus mucosa of Mongolian gerbils infected with Helicobacter pylori. The Journal of pathology. 2016;239(4):399–410. doi: 10.1002/path.4735 27125972 10.1002/path.4735 PMC4958595

[24] Yoo MW, Park J, Han HS, Yun YM, Kang JW, Choi DY, et al Discovery of gastric cancer specific biomarkers by the application of serum proteomics. Proteomics. 2017;17(6). doi: 10.1002/pmic.201600332 28133907 10.1002/pmic.201600332

[25] Weis VG, Petersen CP, Mills JC, Tuma PL, Whitehead RH, Goldenring JR. Establishment of novel in vitro mouse chief cell and SPEM cultures identifies MAL2 as a marker of metaplasia in the stomach. American journal of physiology Gastrointestinal and liver physiology. 2014;307(8):G777–92. doi: 10.1152/ajpgi.00169.2014 25190476 10.1152/ajpgi.00169.2014 PMC4200317

[26] Bakkelund KE, Waldum HL, Nordrum IS, Hauso O, Fossmark R. Long-term gastric changes in achlorhydric H(+)/K(+)-ATPase beta subunit deficient mice. Scandinavian journal of gastroenterology. 2010;45(9):1042–7. doi: 10.3109/00365521.2010.490952 20476858 10.3109/00365521.2010.490952

[27] Sordal O, Waldum H, Nordrum IS, Boyce M, Bergh K, Munkvold B, et al The gastrin receptor antagonist netazepide (YF476) prevents oxyntic mucosal inflammation induced by Helicobacter pylori infection in Mongolian gerbils. Helicobacter. 2013;18(6):397–405. doi: 10.1111/hel.12066 23865485 10.1111/hel.12066

[28] Kleveland PM, Haugen SE, Waldum HL. The preparation of bioactive 125I-gastrin, using Iodo-gen as oxidizing agent, and the use of this tracer in receptor studies. Scandinavian journal of gastroenterology. 1985;20(5):569–76. 2992067 10.3109/00365528509089698

[29] Akbari M, Otterlei M, Pena-Diaz J, Aas PA, Kavli B, Liabakk NB, et al Repair of U/G and U/A in DNA by UNG2-associated repair complexes takes place predominantly by short-patch repair both in proliferating and growth-arrested cells. Nucleic acids research. 2004;32(18):5486–98. doi: 10.1093/nar/gkh872 15479784 10.1093/nar/gkh872 PMC524284

[30] Wessel D, Flugge UI. A method for the quantitative recovery of protein in dilute solution in the presence of detergents and lipids. Anal Biochem. 1984;138(1):141–3. 6731838 10.1016/0003-2697(84)90782-6

[31] MacLean B, Tomazela DM, Shulman N, Chambers M, Finney GL, Frewen B, et al Skyline: an open source document editor for creating and analyzing targeted proteomics experiments. Bioinformatics. 2010;26(7):966–8. doi: 10.1093/bioinformatics/btq054 20147306 10.1093/bioinformatics/btq054 PMC2844992

[32] Liang CC, Park AY, Guan JL. In vitro scratch assay: a convenient and inexpensive method for analysis of cell migration in vitro. Nature protocols. 2007;2(2):329–33. doi: 10.1038/nprot.2007.30 17406593 10.1038/nprot.2007.30

[33] Lenferink AE, Cantin C, Nantel A, Wang E, Durocher Y, Banville M, et al Transcriptome profiling of a TGF-beta-induced epithelial-to-mesenchymal transition reveals extracellular clusterin as a target for therapeutic antibodies. Oncogene. 2010;29(6):831–44. doi: 10.1038/onc.2009.399 19935703 10.1038/onc.2009.399

[34] Solcia E, Bordi C, Creutzfeldt W, Dayal Y, Dayan AD, Falkmer S, et al Histopathological classification of nonantral gastric endocrine growths in man. Digestion. 1988;41(4):185–200. 3072229 10.1159/000199786

[35] Schindelin J, Arganda-Carreras I, Frise E, Kaynig V, Longair M, Pietzsch T, et al Fiji: an open-source platform for biological-image analysis. Nature methods. 2012;9(7):676–82. doi: 10.1038/nmeth.2019 22743772 10.1038/nmeth.2019 PMC3855844

[36] Lennerz JK, Kim SH, Oates EL, Huh WJ, Doherty JM, Tian X, et al The transcription factor MIST1 is a novel human gastric chief cell marker whose expression is lost in metaplasia, dysplasia, and carcinoma. The American journal of pathology. 2010;177(3):1514–33. doi: 10.2353/ajpath.2010.100328 20709804 10.2353/ajpath.2010.100328 PMC2928982

[37] Franic TV, Judd LM, Robinson D, Barrett SP, Scarff KL, Gleeson PA, et al Regulation of gastric epithelial cell development revealed in H(+)/K(+)-ATPase beta-subunit- and gastrin-deficient mice. American journal of physiology Gastrointestinal and liver physiology. 2001;281(6):G1502–11. 11705756 10.1152/ajpgi.2001.281.6.G1502

[38] Scarff KL, Judd LM, Toh BH, Gleeson PA, Van Driel IR. Gastric H(+),K(+)-adenosine triphosphatase beta subunit is required for normal function, development, and membrane structure of mouse parietal cells. Gastroenterology. 1999;117(3):605–18. 10464136 10.1016/s0016-5085(99)70453-1

[39] Nam KT, Lee HJ, Sousa JF, Weis VG, O'Neal RL, Finke PE, et al Mature chief cells are cryptic progenitors for metaplasia in the stomach. Gastroenterology. 2010;139(6):2028–37 e9. doi: 10.1053/j.gastro.2010.09.005 20854822 10.1053/j.gastro.2010.09.005 PMC2997152

[40] Chen X, Leung SY, Yuen ST, Chu KM, Ji J, Li R, et al Variation in gene expression patterns in human gastric cancers. Mol Biol Cell. 2003;14(8):3208–15. doi: 10.1091/mbc.E02-12-0833 12925757 10.1091/mbc.E02-12-0833 PMC181561

[41] Ooi CH, Ivanova T, Wu J, Lee M, Tan IB, Tao J, et al Oncogenic pathway combinations predict clinical prognosis in gastric cancer. PLoS Genet. 2009;5(10):e1000676 doi: 10.1371/journal.pgen.1000676 19798449 10.1371/journal.pgen.1000676 PMC2748685

[42] Forster S, Gretschel S, Jons T, Yashiro M, Kemmner W. THBS4, a novel stromal molecule of diffuse-type gastric adenocarcinomas, identified by transcriptome-wide expression profiling. Mod Pathol. 2011;24(10):1390–403. doi: 10.1038/modpathol.2011.99 21701537 10.1038/modpathol.2011.99

[43] D'Errico M, de Rinaldis E, Blasi MF, Viti V, Falchetti M, Calcagnile A, et al Genome-wide expression profile of sporadic gastric cancers with microsatellite instability. European journal of cancer. 2009;45(3):461–9. doi: 10.1016/j.ejca.2008.10.032 19081245 10.1016/j.ejca.2008.10.032

[44] Bhandari S, Bakke I, Kumar J, Beisvag V, Sandvik AK, Thommesen L, et al Connective tissue growth factor is activated by gastrin and involved in gastrin-induced migration and invasion. Biochemical and biophysical research communications. 2016;475(1):119–24. doi: 10.1016/j.bbrc.2016.05.052 27179776 10.1016/j.bbrc.2016.05.052

[45] Rao SV, Solum G, Niederdorfer B, Norsett KG, Bjorkoy G, Thommesen L. Gastrin activates autophagy and increases migration and survival of gastric adenocarcinoma cells. BMC cancer. 2017;17(1):68 doi: 10.1186/s12885-017-3055-5 28109268 10.1186/s12885-017-3055-5 PMC5251222

[46] Norsett KG, Steele I, Duval C, Sammut SJ, Murugesan SV, Kenny S, et al Gastrin stimulates expression of plasminogen activator inhibitor-1 in gastric epithelial cells. American journal of physiology Gastrointestinal and liver physiology. 2011;301(3):G446–53. doi: 10.1152/ajpgi.00527.2010 21193525 10.1152/ajpgi.00527.2010 PMC3174540

[47] Yang W, Soares J, Greninger P, Edelman EJ, Lightfoot H, Forbes S, et al Genomics of Drug Sensitivity in Cancer (GDSC): a resource for therapeutic biomarker discovery in cancer cells. Nucleic acids research. 2013;41(Database issue):D955–61. doi: 10.1093/nar/gks1111 23180760 10.1093/nar/gks1111 PMC3531057

[48] Bringeland EA, Wasmuth HH, Fougner R, Mjones P, Gronbech JE. Impact of perioperative chemotherapy on oncological outcomes after gastric cancer surgery. The British journal of surgery. 2014;101(13):1712–20. doi: 10.1002/bjs.9650 25312592 10.1002/bjs.9650

[49] Andersen CL, Schepeler T, Thorsen K, Birkenkamp-Demtroder K, Mansilla F, Aaltonen LA, et al Clusterin expression in normal mucosa and colorectal cancer. Molecular & cellular proteomics: MCP. 2007;6(6):1039–48. 17322305 10.1074/mcp.M600261-MCP200

[50] Henderson-Jackson EB, Nasir A, Chen DT, Nandyala P, Djeu J, Strosberg J, et al Cytoplasmic Clusterin expression correlates with pancreatic neuroendocrine tumor size and pathological stage. Pancreas. 2013;42(6):967–70. doi: 10.1097/MPA.0b013e318293734b 23770713 10.1097/MPA.0b013e318293734b PMC4644941

[51] Mourra N, Scriva A, Mansiaux Y, Gozlan S, Bennis M, Balaton A. Clusterin expression in gastrointestinal neuroendocrine tumours is highly correlated with location and is helpful in determining the origin of liver metastases. Histopathology. 2014;65(5):642–50. doi: 10.1111/his.12450 24807631 10.1111/his.12450

[52] Selvik LK, Fjeldbo CS, Flatberg A, Steigedal TS, Misund K, Anderssen E, et al The duration of gastrin treatment affects global gene expression and molecular responses involved in ER stress and anti-apoptosis. BMC genomics. 2013;14:429 doi: 10.1186/1471-2164-14-429 23805861 10.1186/1471-2164-14-429 PMC3698217

[53] Hayakawa Y, Jin G, Wang H, Chen X, Westphalen CB, Asfaha S, et al CCK2R identifies and regulates gastric antral stem cell states and carcinogenesis. Gut. 2015;64(4):544–53. doi: 10.1136/gutjnl-2014-307190 24951258 10.1136/gutjnl-2014-307190 PMC4627594

[54] Dimaline R, Varro A. Novel roles of gastrin. The Journal of physiology. 2014;592(Pt 14):2951–8. 24665102 10.1113/jphysiol.2014.272435 PMC4214651

[55] Hayakawa Y, Chang W, Jin G, Wang TC. Gastrin and upper GI cancers. Curr Opin Pharmacol. 2016;31:31–7. doi: 10.1016/j.coph.2016.08.013 27591354 10.1016/j.coph.2016.08.013

[56] Gutacker C, Klock G, Diel P, Koch-Brandt C. Nerve growth factor and epidermal growth factor stimulate clusterin gene expression in PC12 cells. The Biochemical journal. 1999;339 (Pt 3):759–66. 10215617 PMC1220214

[57] Franic TV, van Driel IR, Gleeson PA, Giraud AS, Judd LM. Reciprocal changes in trefoil 1 and 2 expression in stomachs of mice with gastric unit hypertrophy and inflammation. The Journal of pathology. 2005;207(1):43–52. doi: 10.1002/path.1811 15983982 10.1002/path.1811

[58] Howlett M, Giraud AS, Lescesen H, Jackson CB, Kalantzis A, Van Driel IR, et al The interleukin-6 family cytokine interleukin-11 regulates homeostatic epithelial cell turnover and promotes gastric tumor development. Gastroenterology. 2009;136(3):967–77. doi: 10.1053/j.gastro.2008.12.003 19121317 10.1053/j.gastro.2008.12.003

[59] Burclaff J, Osaki LH, Liu D, Goldenring JR, Mills JC. Targeted Apoptosis of Parietal Cells Is Insufficient to Induce Metaplasia in Stomach. Gastroenterology. 2017;152(4):762–6 e7. doi: 10.1053/j.gastro.2016.12.001 27932312 10.1053/j.gastro.2016.12.001 PMC5391042

[60] Nomura S, Yamaguchi H, Ogawa M, Wang TC, Lee JR, Goldenring JR. Alterations in gastric mucosal lineages induced by acute oxyntic atrophy in wild-type and gastrin-deficient mice. American journal of physiology Gastrointestinal and liver physiology. 2005;288(2):G362–75. doi: 10.1152/ajpgi.00160.2004 15647607 10.1152/ajpgi.00160.2004

[61] Takaishi S, Cui G, Frederick DM, Carlson JE, Houghton J, Varro A, et al Synergistic inhibitory effects of gastrin and histamine receptor antagonists on Helicobacter-induced gastric cancer. Gastroenterology. 2005;128(7):1965–83. 15940630 10.1053/j.gastro.2005.03.027

[62] Takaishi S, Tu S, Dubeykovskaya ZA, Whary MT, Muthupalani S, Rickman BH, et al Gastrin is an essential cofactor for helicobacter-associated gastric corpus carcinogenesis in C57BL/6 mice. The American journal of pathology. 2009;175(1):365–75. doi: 10.2353/ajpath.2009.081165 19556515 10.2353/ajpath.2009.081165 PMC2708822

[63] Seishima R, Wada T, Tsuchihashi K, Okazaki S, Yoshikawa M, Oshima H, et al Ink4a/Arf-Dependent Loss of Parietal Cells Induced by Oxidative Stress Promotes CD44-Dependent Gastric Tumorigenesis. Cancer Prev Res (Phila). 2015;8(6):492–501. 25813526 10.1158/1940-6207.CAPR-15-0025-T

[64] Kim JP, Kim SC, Yang HK. Prognostic significance of signet ring cell carcinoma of the stomach. Surg Oncol. 1994;3(4):221–7. 7834113 10.1016/0960-7404(94)90037-x

[65] Taghavi S, Jayarajan SN, Davey A, Willis AI. Prognostic significance of signet ring gastric cancer. J Clin Oncol. 2012;30(28):3493–8. doi: 10.1200/JCO.2012.42.6635 22927530 10.1200/JCO.2012.42.6635 PMC3454770

[66] Zhang M, Zhu G, Zhang H, Gao H, Xue Y. Clinicopathologic features of gastric carcinoma with signet ring cell histology. Journal of gastrointestinal surgery: official journal of the Society for Surgery of the Alimentary Tract. 2010;14(4):601–6. 20033340 10.1007/s11605-009-1127-9

[67] Zhang Y, Chen JN, Dong M, Zhang ZG, Zhang YW, Wu JY, et al Clinical significance of spasmolytic polypeptide-expressing metaplasia and intestinal metaplasia in Epstein-Barr virus-associated and Epstein-Barr virus-negative gastric cancer. Human pathology. 2017;63:128–38. doi: 10.1016/j.humpath.2017.02.016 28300576 10.1016/j.humpath.2017.02.016

[68] Yamaguchi H, Goldenring JR, Kaminishi M, Lee JR. Identification of spasmolytic polypeptide expressing metaplasia (SPEM) in remnant gastric cancer and surveillance postgastrectomy biopsies. Digestive diseases and sciences. 2002;47(3):573–8. 11911345 10.1023/a:1017920220149

[69] Nozaki K, Ogawa M, Williams JA, Lafleur BJ, Ng V, Drapkin RI, et al A molecular signature of gastric metaplasia arising in response to acute parietal cell loss. Gastroenterology. 2008;134(2):511–22. doi: 10.1053/j.gastro.2007.11.058 18242217 10.1053/j.gastro.2007.11.058 PMC2857727

[70] Zhang F, Kumano M, Beraldi E, Fazli L, Du C, Moore S, et al Clusterin facilitates stress-induced lipidation of LC3 and autophagosome biogenesis to enhance cancer cell survival. Nature communications. 2014;5:5775 doi: 10.1038/ncomms6775 25503391 10.1038/ncomms6775 PMC4275590

[71] Alnasser HA, Guan Q, Zhang F, Gleave ME, Nguan CY, Du C. Requirement of clusterin expression for prosurvival autophagy in hypoxic kidney tubular epithelial cells. Am J Physiol Renal Physiol. 2016;310(2):F160–73. doi: 10.1152/ajprenal.00304.2015 26561650 10.1152/ajprenal.00304.2015

[72] Trougakos IP, Lourda M, Antonelou MH, Kletsas D, Gorgoulis VG, Papassideri IS, et al Intracellular clusterin inhibits mitochondrial apoptosis by suppressing p53-activating stress signals and stabilizing the cytosolic Ku70-Bax protein complex. Clinical cancer research: an official journal of the American Association for Cancer Research. 2009;15(1):48–59. 19118032 10.1158/1078-0432.CCR-08-1805 PMC4483278

[73] Zhang H, Kim JK, Edwards CA, Xu Z, Taichman R, Wang CY. Clusterin inhibits apoptosis by interacting with activated Bax. Nature cell biology. 2005;7(9):909–15. doi: 10.1038/ncb1291 16113678 10.1038/ncb1291

