# Supplementary figures and images for: The cytoprotective protein clusterin is overexpressed in hypergastrinemic rodent models of oxyntic preneoplasia and promotes gastric cancer cell survival
Source: PLoS One. 2017 Sep 13;12(9):e0184514. doi: 10.1371/journal.pone.0184514 (PMC5597207; doi:10.1371/journal.pone.0184514)

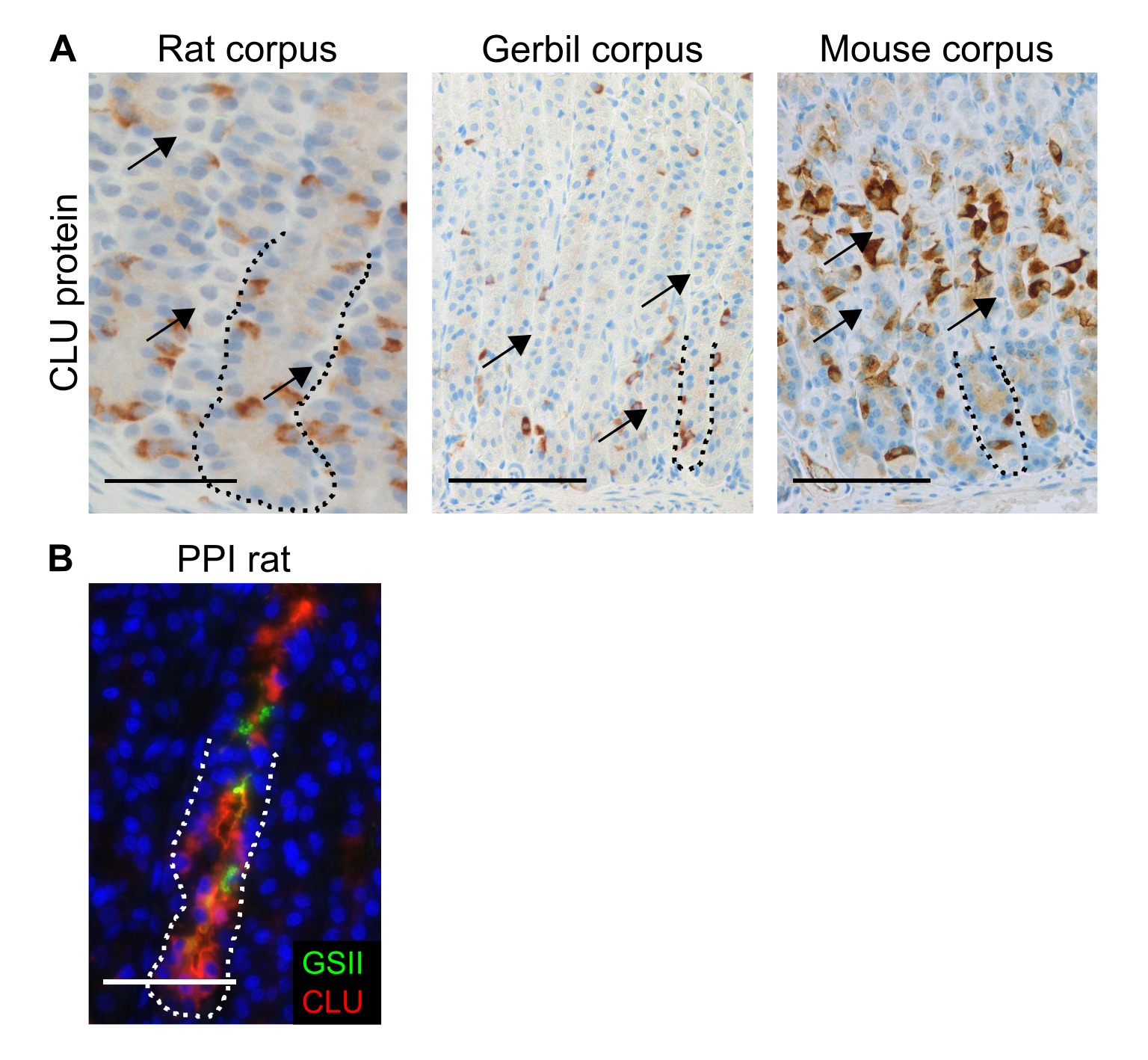

Supplement: S1 Fig — (A) IHC of oxyntic/corpus mucosa from control rat, Mongolian gerbil and mouse showing no expression of CLU (brown) in parietal cells (arrows). (B) Double immunofluorescence staining of oxyntic mucosa from hypergastrinemic PPI-rats showing CLU (red) expression in a few mucous neck cells (GSII-positive (green)) in the lower neck region of oxyntic glands. Nuclei were counterstained with hematoxylin (blue) or DAPI (blue). The basal zone (~100 μm from the gland bottom) is highlighted with a dotted line. Scale bars (A middle and right column) = 100 μm; (A left column, B) 50 μm. (TIF) [file pone.0184514.s001.tif]

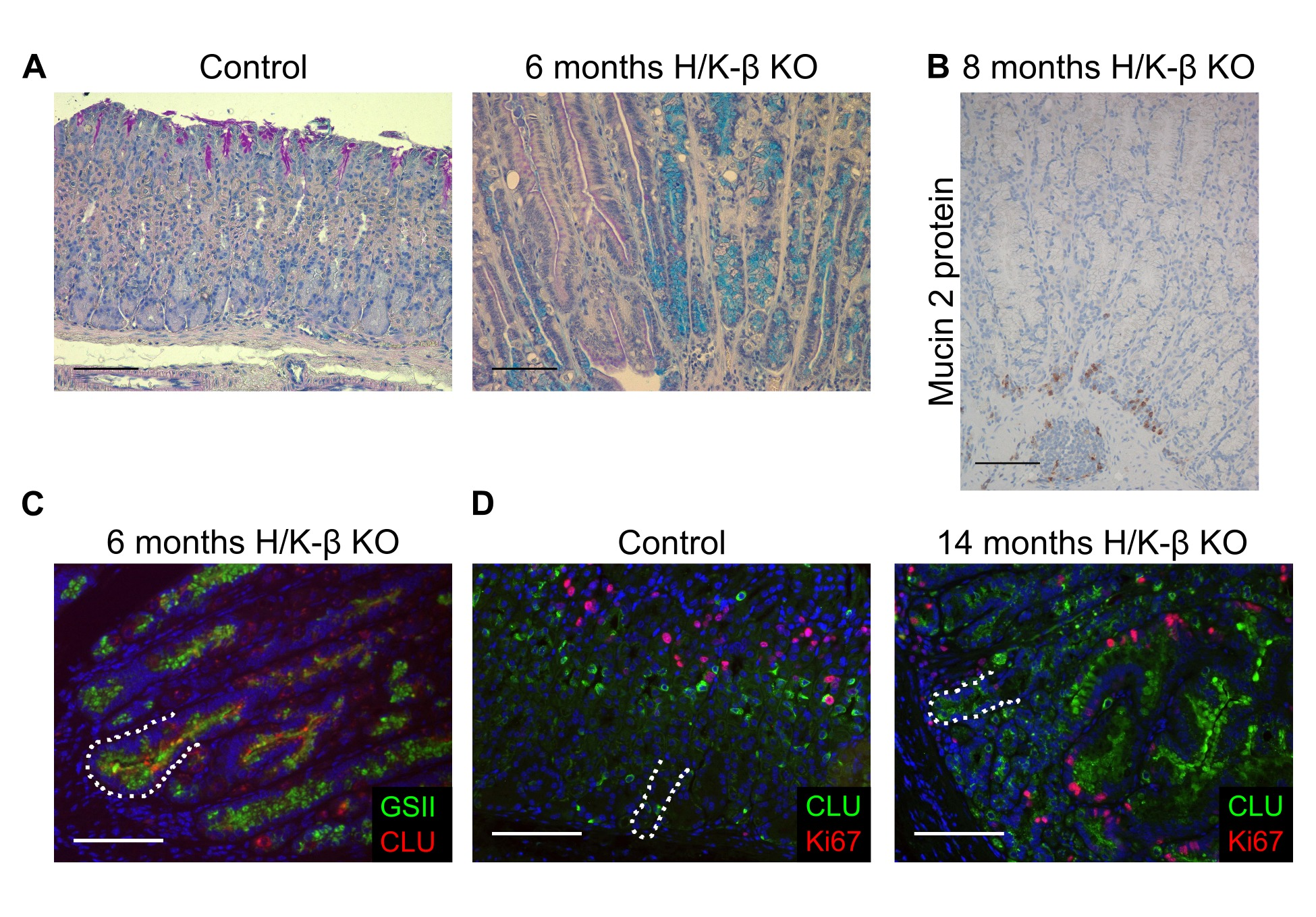

Supplement: S2 Fig — (A) Periodic acid Schiff and Alcian blue staining of oxyntic mucosa from wild-type control and H/K-β KO mice aged 6 months. (B) IHC staining of oxyntic mucosa from H/K-β KO mice aged 8 months showing no mucin 2 expression in the gastric epithelial cells. There is some positivity in scattered single cells in the underlying stroma. (C) Double immunofluorescence staining of oxyntic mucosa from H/K-β KO mice aged 6 months showing CLU (red) expression in GSII-positive cells (green). (D) Double immunofluorescence staining of oxyntic mucosa from wild-type control mice and H/K-β KO mice aged 14 months showing co-expression of CLU (green) and the proliferation marker Ki67 (red). Nuclei were counterstained with hematoxylin (blue) or DAPI (blue). The basal zone (~100 μm from the gland bottom) is highlighted with a dotted line. Scale bars = 100 μm. (TIF) [file pone.0184514.s002.tif]

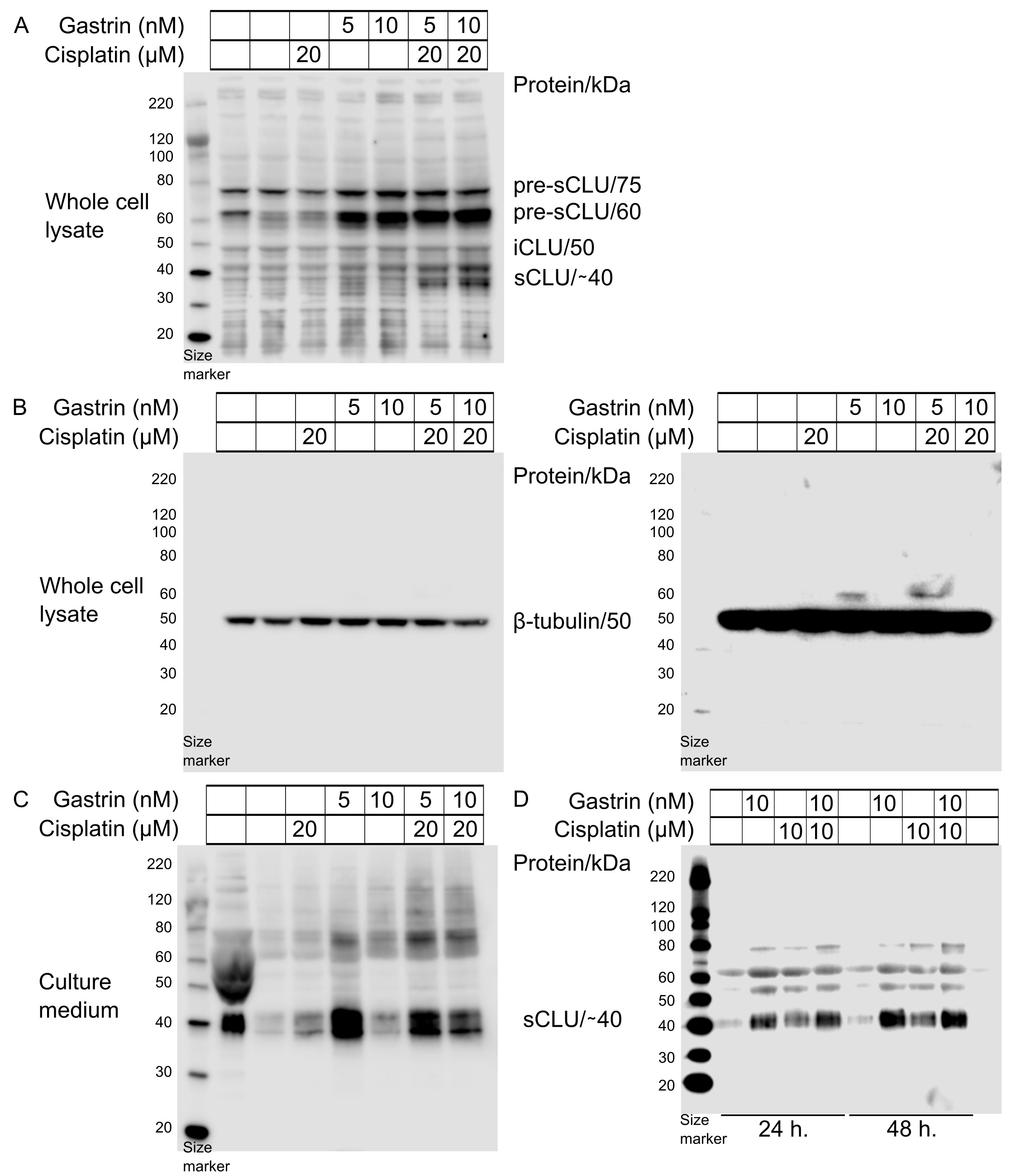

Supplement: S3 Fig — (A) Western blot showing that gastrin and/or cisplatin for 24 hours stimulated increased expression of CLU in AGS-GR cells. (B) β-tubulin was used as loading control. Left image is shown with low contrast. Right image is shown with high contrast, in order to visualize the molecular size marker. (C) Western blot of the pertaining culture medium showing that gastrin and/or cisplatin for 24 hours stimulated increased secretion of sCLU from AGS-GR cells. (D) Due to a technical issue with the lane for gastrin 10 nM in (C), resulting in weak signals, we demonstrate an additional gel blot, from an independent experiment, showing that gastrin and/or cisplatin for 24 or 48 hours stimulated increased secretion of sCLU from AGS-GR cells. pre-sCLU = precursor of secretory CLU; iCLU = intracellular CLU; sCLU = secretory CLU. (TIF) [file pone.0184514.s003.tif]

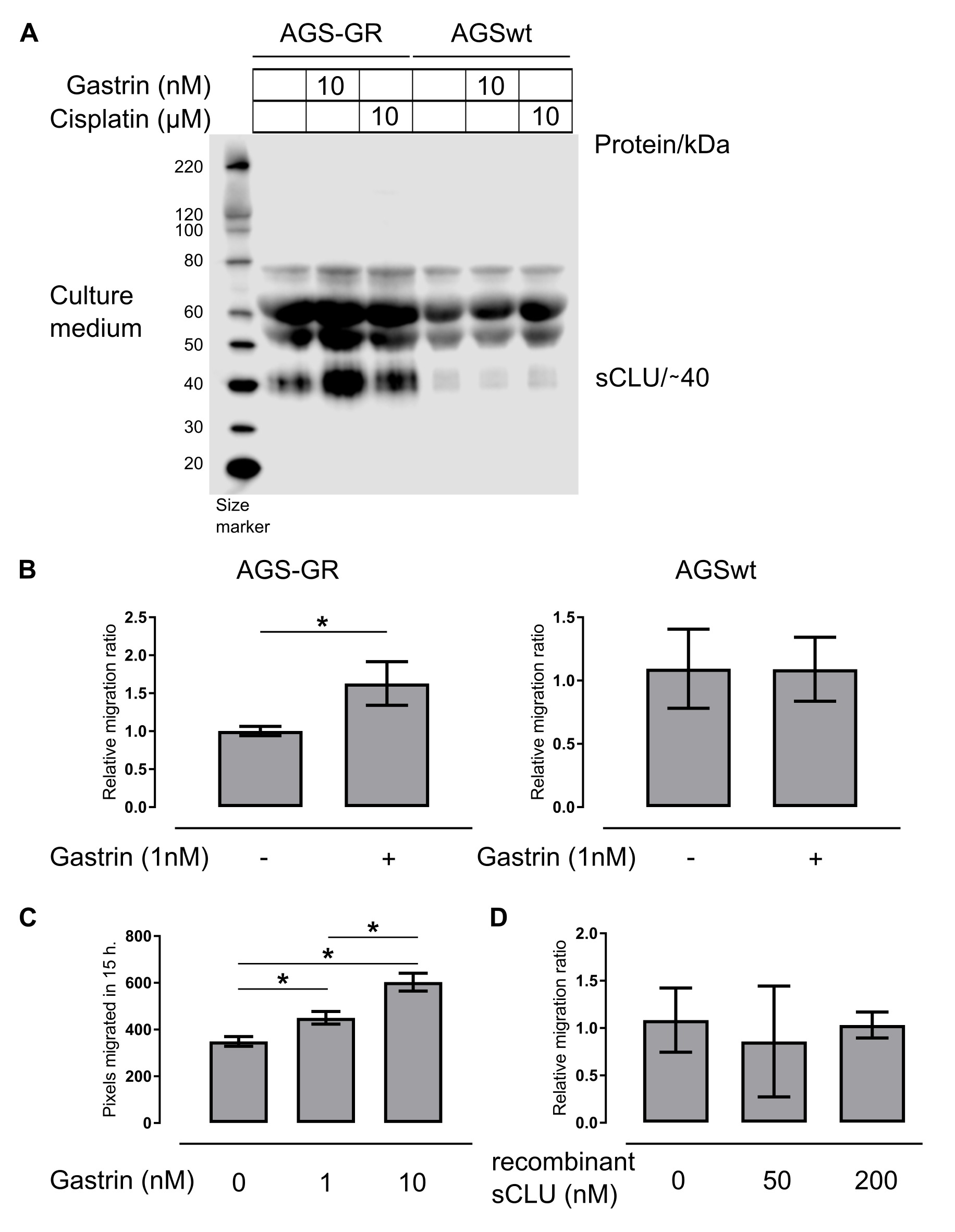

Supplement: S4 Fig — (A) Western blot of culture medium showing that gastrin or cisplatin for 48 hours stimulated increased secretion of sCLU in AGS-GR and not AGS wild type (AGSwt) cells. sCLU = secretory CLU. (B) Quantification of migration of AGS-GR and AGSwt cells after 18 hours in the absence or presence of gastrin 1 nM. Relative migration ratio was estimated using data from 5 independent experiments (2–7 technical replicates in each experiment) with AGS-GR and 2 independent experiments (3–5 technical replicates in each experiment) with AGSwt. (C) Quantification of migration, as number of pixels migrated, of AGS-GR cells in a scratch assay after 15 hours in the absence or presence of gastrin 1 nM or 10 nM. Data from 3 independent experiments. (D) Quantification of migration of AGS-GR cells after 18 hours in the presence of recombinant sCLU at 50 or 200 nM. Relative migration ratio was estimated using data from 2 independent experiments (2–3 technical replicates in each experiment). Data was normalized to the median cell index of untreated cells in each independent experiment. Data is presented as means with error bars representing 95% confidence intervals. *Students t-test with Bonferroni-adjusted p value < 0.05. (TIF) [file pone.0184514.s004.tif]

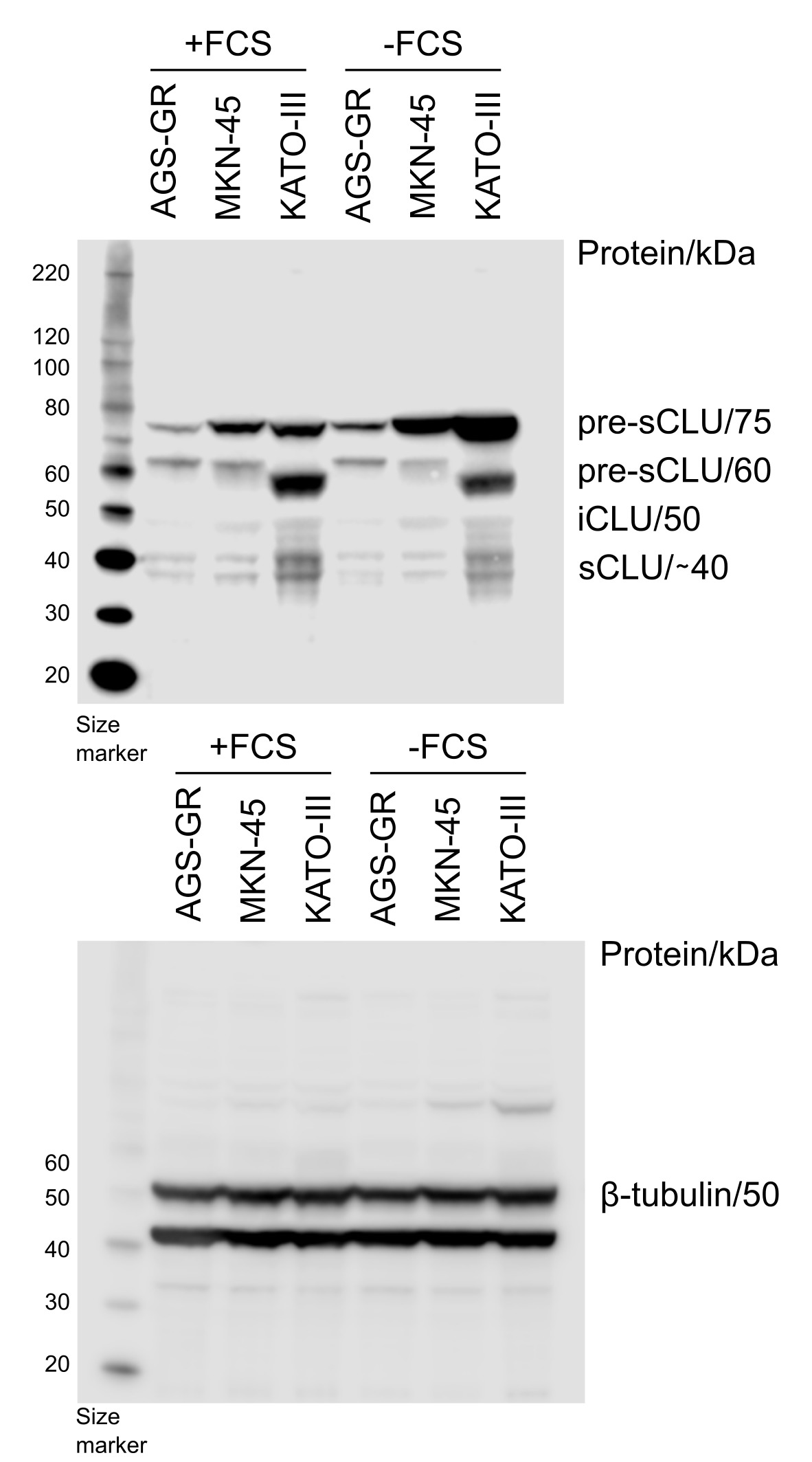

Supplement: S5 Fig — Western blot showing different isoforms of CLU expressed in the gastric cancer cell lines AGS-GR, MKN-45 and KATO-III, cultured with and without fetal calf serum (FCS). β-tubulin was used as loading control. pre-sCLU = precursor of secretory CLU; iCLU = intracellular CLU; sCLU = secretory CLU. (TIF) [file pone.0184514.s005.tif]

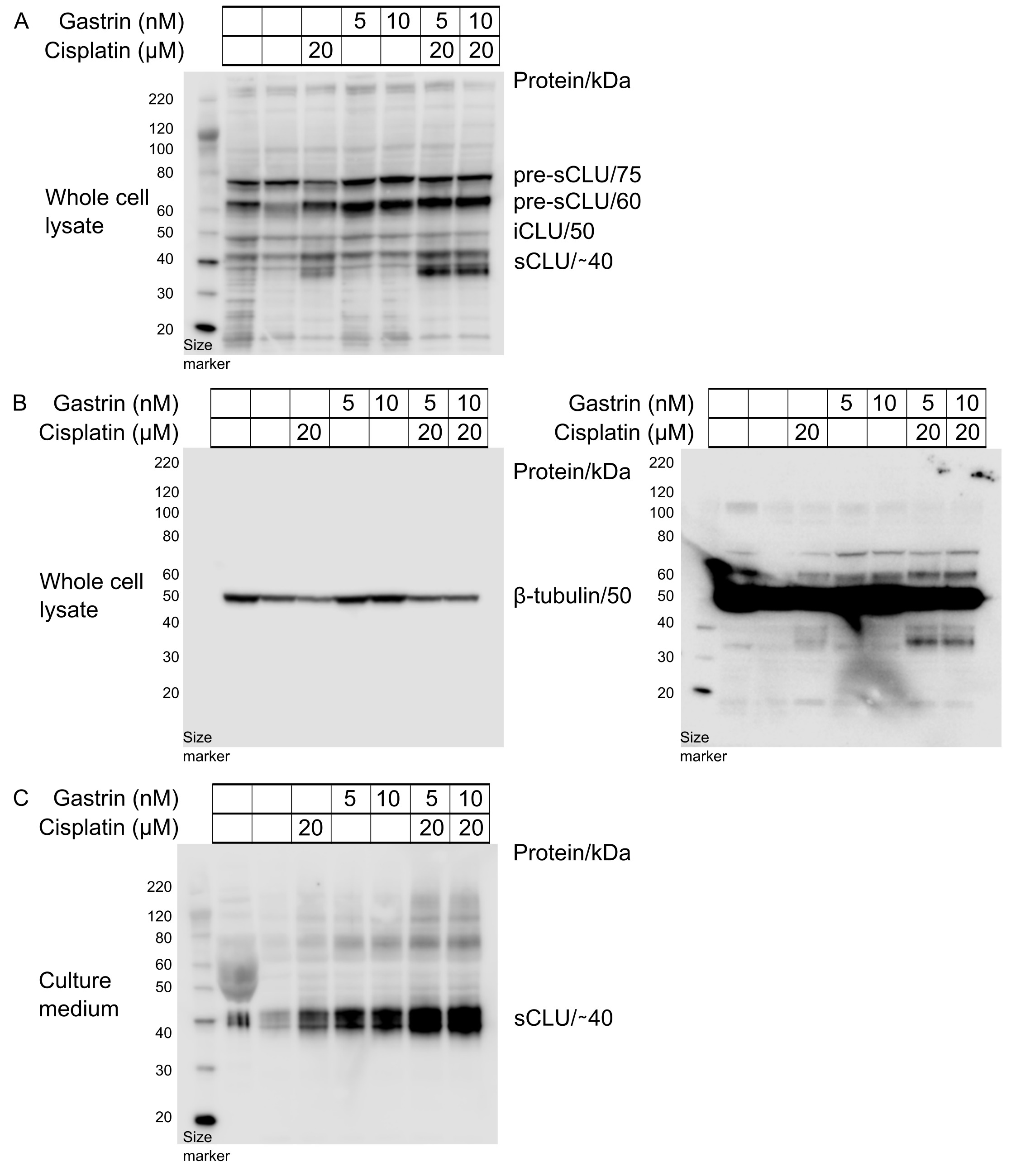

Supplement: S6 Fig — (A) Western blot showing that gastrin and/or cisplatin for 48 hours stimulated increased expression of CLU in AGS-GR cells. (B) β-tubulin was used as loading control. Left image is shown with low contrast. Right image is shown with high contrast, in order to visualize the molecular size marker. (C) Western blot of the pertaining culture medium showing that gastrin and/or cisplatin for 48 hours stimulated increased secretion of sCLU from AGS-GR cells. pre-sCLU = precursor of secretory CLU; iCLU = intracellular CLU; sCLU = secretory CLU. (TIF) [file pone.0184514.s006.tif]

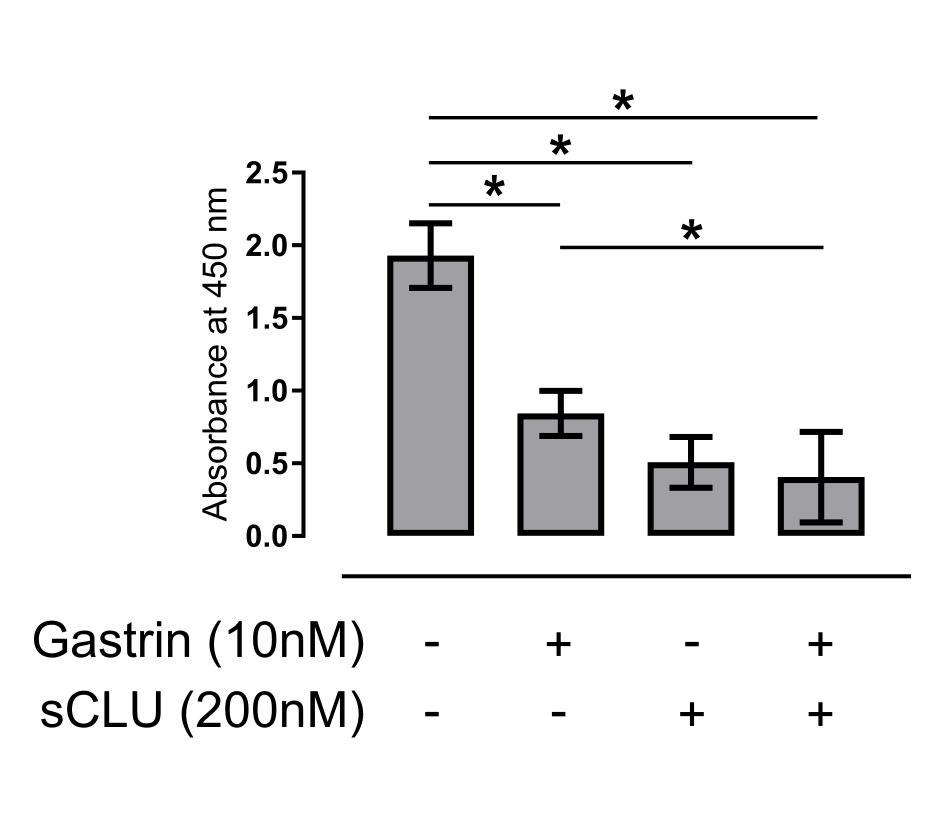

Supplement: S7 Fig — Apoptosis was induced by serum-starvation for 72 hours and TUNEL staining quantified as absorbance at 450 nm in AGS-GR cells grown in the absence or presence of gastrin (10 nM), sCLU (200 nM), or both. A positive control with nuclease treatment of cells showed absorbance 3.67, and a negative control with omission of the reaction enzyme showed absorbance 0.27. Data is presented as mean of 6 technical replicates with error bars representing 95% confidence intervals. Results are representative for two independent experiments (6 technical replicates in each experiment). *ANOVA with Tukey-adjusted p value < 0.05. (TIF) [file pone.0184514.s007.tif]
